# A fish herpesvirus highlights functional diversities among Zα domains related to phase separation induction and A-to-Z conversion

**DOI:** 10.1093/nar/gkac761

**Published:** 2022-09-22

**Authors:** Mamadou Amadou Diallo, Sébastien Pirotte, Yunlong Hu, Léa Morvan, Krzysztof Rakus, Nicolás M Suárez, Lee PoTsang, Hisao Saneyoshi, Yan Xu, Andrew J Davison, Peter Tompa, Joel L Sussman, Alain Vanderplasschen

**Affiliations:** Department of Infectious and Parasitic Diseases, Immunology-Vaccinology, University of Liège, Liège B-4000, Belgium; Department of Infectious and Parasitic Diseases, Immunology-Vaccinology, University of Liège, Liège B-4000, Belgium; Department of Infectious and Parasitic Diseases, Immunology-Vaccinology, University of Liège, Liège B-4000, Belgium; Department of Infectious and Parasitic Diseases, Immunology-Vaccinology, University of Liège, Liège B-4000, Belgium; Department of Infectious and Parasitic Diseases, Immunology-Vaccinology, University of Liège, Liège B-4000, Belgium; Department of Evolutionary Immunology, Institute of Zoology and Biomedical Research, Faculty of Biology, Jagiellonian University, Krakow 30387, Poland; MRC-University of Glasgow Centre for Virus Research, Glasgow G61 1QH, UK; Department of Infectious and Parasitic Diseases, Immunology-Vaccinology, University of Liège, Liège B-4000, Belgium; Department of Aquaculture, National Taiwan Ocean University, Keelung 202, Taiwan; Department of Medical Sciences, Division of Chemistry, University of Miyazaki, Miyazaki 889-1692, Japan; Department of Medical Sciences, Division of Chemistry, University of Miyazaki, Miyazaki 889-1692, Japan; MRC-University of Glasgow Centre for Virus Research, Glasgow G61 1QH, UK; VIB-VUB Center for Structural Biology, Vrije Universiteit Brussel, Brussel B-1050, Belgium; Department of Chemical and Structural Biology, Weizmann Institute of Science, Rehovot 7610001, Israel; Department of Infectious and Parasitic Diseases, Immunology-Vaccinology, University of Liège, Liège B-4000, Belgium

## Abstract

Zalpha (Zα) domains bind to left-handed Z-DNA and Z-RNA. The Zα domain protein family includes cellular (ADAR1, ZBP1 and PKZ) and viral (vaccinia virus E3 and cyprinid herpesvirus 3 (CyHV-3) ORF112) proteins. We studied CyHV-3 ORF112, which contains an intrinsically disordered region and a Zα domain. Genome editing of CyHV-3 indicated that the expression of only the Zα domain of ORF112 was sufficient for normal viral replication in cell culture and virulence in carp. In contrast, its deletion was lethal for the virus. These observations revealed the potential of the CyHV-3 model as a unique platform to compare the exchangeability of Zα domains expressed alone in living cells. Attempts to rescue the ORF112 deletion by a broad spectrum of cellular, viral, and artificial Zα domains showed that only those expressing Z-binding activity, the capacity to induce liquid-liquid phase separation (LLPS), and A-to-Z conversion, could rescue viral replication. For the first time, this study reports the ability of some Zα domains to induce LLPS and supports the biological relevance of dsRNA A-to-Z conversion mediated by Zα domains. This study expands the functional diversity of Zα domains and stimulates new hypotheses concerning the mechanisms of action of proteins containing Zα domains.

## Graphical Abstract

**Graphical Abstract:**
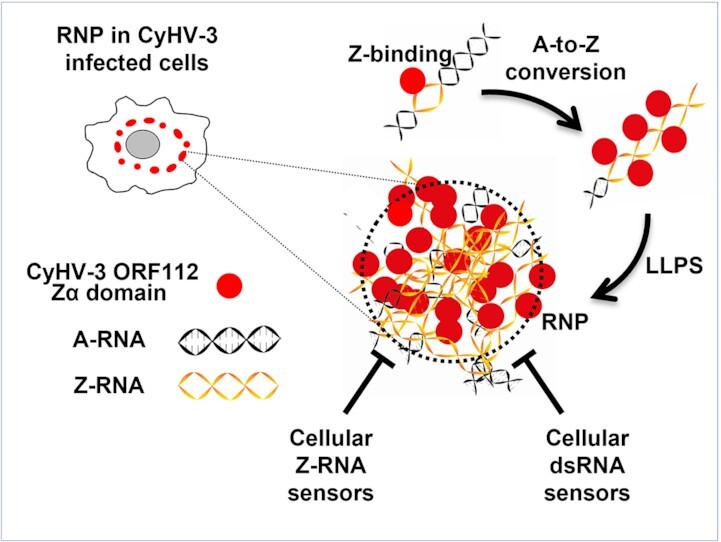
Schematic model linking biochemical properties of Zα domains and the formation of RNP observed in CyHV-3 infected cells.

## INTRODUCTION

Double-stranded (ds) RNA and dsDNA exist under two common structural conformations named A and B. These conformations are both right-handed helices and represent the predominant form of dsRNA and dsDNA in cells, respectively. Other less common conformations of dsDNA and dsRNA have also been reported, such as the Z conformation with a left-handed structure (1). Z-RNA and Z-DNA have been reported in a growing list of physiological and pathological processes such as transcriptional regulation (2,3), innate immunity (4,5), and viral infection (6). It has been shown *in vitro* that the Z-conformation of DNA is inherently metastable, indicating that it is ideally suited to act as a structural regulatory element. Such an element should be endowed with a high susceptibility to cellular parameters (7).

**Figure 1.**
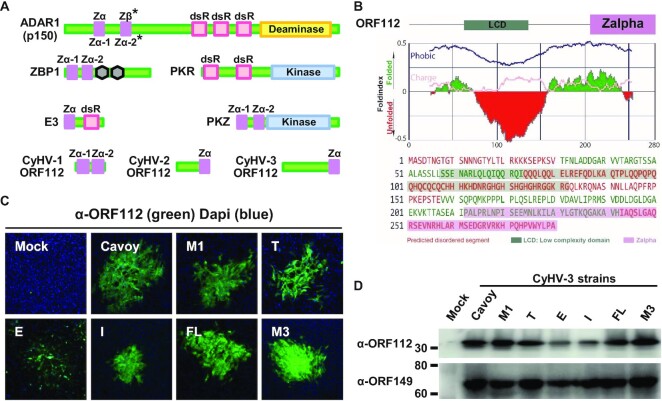
The ORF112 protein is conserved among CyHV-3 strains and consists of an N-terminal intrinsically disordered region and a C-terminal Zα domain. (**A**) Schematic organization of cellular and viral proteins containing Zα domains or right-handed dsRNA-binding domains. Zα, Zalpha domain; dsR, right-handed dsRNA-binding domain; RHIM, RIP homotypic interaction motifs represented by hexagons. * Hs ADAR1 p150 encodes a Zα and a Zβ domains in contrast to teleost ADAR1 orthologues which contains two Zα domains. (**B**) ORF112 sequence analysis by FoldIndex (prediction of disordered regions). (**C**) Immunofluorescent detection of ORF112 in CCB cells infected by the indicated strains of CyHV-3. Fixed and permeabilised cells were immunostained with α-ORF112 antibodies and Alexa Fluor 488 goat anti-rabbit antibodies used as primary and secondary antibodies, respectively. The dimensions of each photograph correspond to 636 μm of the specimen. (**D**) Western blot analysis of cells infected by the indicated CyHV-3 strains. CCB cells grown in six-well plates were mock-infected (Mock) or infected with the indicated strains of CyHV-3 at the MOI of 1 PFU/cell. Twenty-four hours postinfection, cells were lysed and analysed by western blot using α-ORF112 and α-ORF149 as primary antibodies.

Zalpha (Zα) domains bind to left-handed Z-DNA and Z-RNA. They have been reported in only three cellular and two viral proteins (Figure 1A). Cellular proteins that contain Zα domains belong to the innate immune system: the A-to-I RNA-editing enzyme ADAR1 (ADAR1) (found in metazoans) plays a key role in innate antiviral immunity and in preventing innate immune sensing of self-RNA by cytosolic receptors detecting right-handed dsRNA (8,9); the Z-DNA-binding protein 1 (ZBP1) (found in mammals and reptiles but not teleosts) has been shown to induce necroptosis when sensing viral and endogenous Z-RNA (10); the protein kinase containing Z-DNA binding domain (PKZ) (found in salmoniforms and cypriniforms) is a paralogue of the dsRNA-dependent protein kinase (PKR) (found in vertebrates) (11). Zα domains have also been reported in two viral proteins (Figure 1A): the E3 protein encoded by vaccinia virus (Vv) (6) and other chordopoxviruses, and the ORF112 protein encoded by cyprinid herpesviruses (CyHV-1, CyHV-2 and CyHV-3) (12). The E3 Zα domain of Vv has been shown to form condensates with dsRNA and Z-RNA in infected cells and to inhibit the detection of Z-RNA by ZBP1 and subsequent necroptosis of infected cells (6). It is not essential for viral growth in cell culture but is required for virulence in animal models (13,14). Based on the results of gel mobility assays, it has been suggested that the Zα domain of CyHV-3 (a pathogen of common and koi carp) ORF112 may prevent the detection of Z-nucleic acids by PKZ in infected cells (12). CyHV-3 ORF112 is the subject of the present study.

Zα domain-containing proteins are multidomain proteins. Zα domains expressed alone have been studied at the structural, biochemical, and biological levels. NMR and crystallographic studies (15,16) have demonstrated that different Zα domains consist of a short-winged helix-turn-helix that bind similarly to left-handed nucleic acids, suggesting that they are functionally equivalent. However, other biochemical experiments have demonstrated their functional diversity in terms of dsDNA recognition (B *versus* Z) (17,18). In addition to the affinity for Z-DNA common to all Zα domains, a subgroup of Zα domains with structural flexibility (17) or enrichment in positively charged amino acids in their β-wing (19) expressed affinity to B-DNA. The latter property is a crucial step in B-to-Z DNA conversion mediated by some Zα domains. NMR studies led to the classification of Zα domains as non-converters, poor-converters or good-converters (11,18). This functional diversity has been demonstrated only in cell-free assays and never in intact cells (20–22). Only a very few papers reported on studies of Zα domains expressed alone in cells. Arsenite treatment of cells ectopically expressing Zα domains indicated that all tested Zα domains (including the Zα domain of CyHV-3 ORF112) localized in induced stress granules (SG) (23–25). This recruitment was shown to depend on Z-binding based on the testing of different Zα domains (25). This property has never been tested for the Zα domain of CyHV-3 ORF112.

An elegant approach to compare the biological properties of Zα domains under cellular physiological conditions consists of testing the exchangeability of Zα domains between different Zα domain-containing proteins. However, very few studies have adopted this approach, and these have suggested that Zα domains are functionally equivalent (6,14,26). This conclusion relied on experiments performed on Vv E3. The E3 Zα domain (which has only Z-binding activity) was replaced by Zα domains from *Homo sapiens* (Hs) ADAR1 (Zα or Zβ-I335Y (in which Y is restored)) or ZBP1 (Zα-1), generating recombinant strains comparable to the parental strain in cell culture and *in vivo* (6,14,26).

Here, we have studied the Zα domain of CyHV-3 ORF112. Genome editing of CyHV-3 demonstrated that expression of only the Zα domain of ORF112 was sufficient for normal viral replication in cell culture and virulence in carp. These observations revealed the potential of the CyHV-3 model as a unique platform to compare in living cells the exchangeability of Zα domains expressed alone. Attempts to rescue ORF112 deletion by a broad spectrum of cellular, viral, and artificial (resulting from rational protein engineering) Zα domains indicated that only those expressing Z-binding activity, the capacity to induce liquid-liquid phase separation (LLPS), and A-to-Z conversion, could rescue viral replication. This study is the first to report the ability of some Zα domains to induce LLPS and to show the biological relevance of dsRNA A-to-Z conversion mediated by Zα domains. It expands the functional diversity of Zα domains and stimulates new hypotheses concerning the mechanisms of action of Zα domain-containing proteins.

## MATERIALS AND METHODS

### Cells and CyHV-3 strains

HeLa cells were cultured as recommended by the ATCC. *Cyprinus carpio* brain (CCB) cells were cultured as described previously (27). The CyHV-3 FL strain was isolated in Belgium from a fish that had died from CyHV-3 infection and had been used to produce the FL BAC plasmid (27). Other CyHV-3 strains from different geographical origins were also used (28): the M1 and M3 strains from Belgium; the E strain from England (kindly provided by Dr K. Way, CEFAS); the T strain from Taiwan (kindly provided by Dr S. Bergmann, Friedrich-Loeffler Institute); and the I strain from Israel. The attenuated Cavoy strain from Israel was amplified from a CyHV-3 attenuated vaccine that was commercialized for a short period in the USA (29).

### Primary antibodies

Mouse IgG_2a_ monoclonal antibody (mAb) against CyHV-3 ORF149 (α-ORF149) was kindly provided by Dr W. Fuchs (Friedrich-Loeffler Institute). Rabbit polyclonal antibodies (pAbs) were produced against the synthesized Zα domain of ORF112 (aa189-280). These antibodies are hereafter called α-ORF112. The following commercially available antibodies were also used: rabbit pAbs raised against the N-terminus of Hs ZBP1 (α-ZBP1) (Lifespan BioScience, LS-C297028), mAb 3A2 (IgG_1_) raised against Human antigen R (HuR, also known as ELAV-Like RNA Binding Protein 1) (α-HuR) (Santa Cruz Biotechnologies, Sc-5261), mAb 56-Y (IgG_2a_) raised against Human mRNA-decapping enzyme 1a (Dcp1a) (α-Dcp1a) (Santa Cruz Biotechnologies, Sc-100706), mAb G-8 (IgG_1_) raised against Human fibrillarin (α-Fibrillarin) (Santa Cruz Biotechnologies, Sc-374022), mAb Z22 (IgG_2b_) raised against Z-DNA and Z-RNA (α-Z-RNA) (Absolute antibody, Ab00783-3.0) and mAb J2 (IgG_2a_) (Scicons, 10010500) raised against dsRNA (α-dsRNA).

### Western blotting

Cell monolayers were lysed using radioimmunoprecipitation assay buffer (RIPA buffer) supplemented with protease inhibitors (cOmplete, Sigma-Aldrich, 11836170001). Protein samples were mixed with denaturing Laemmli buffer containing β-mercaptoethanol, incubated for 5 min at 95°C and resolved by SDS-PAGE. Protein transfer to the PVDF membrane was carried out using the trans-blot turbo kit (Bio-Rad, 1704272). Blocking was performed in TBST (20 mM Tris, 150 mM NaCl, 0.1% (v/v) Tween 20) containing 5% (w/v) non-fat dry milk. Incubation with primary α-ORF112 or α-ORF149 antibodies was performed in the blocking buffer. After repeated washes with TBST, membranes were incubated with the appropriate secondary antibodies (Dako goat anti-rabbit-HRP, P0448 or rabbit anti-mouse-HRP, P0260) (dilution of 1:1000) for 2 h at room temperature. Chemiluminescence detection was performed after the addition of substrate (SuperSignal West Pico chemiluminescence, Thermo Fisher Scientific, 34580).

### Production of the CyHV-3 BAC ORF112 KO recombinant plasmid using BAC cloning and prokaryotic recombination technologies

**Figure 2.**
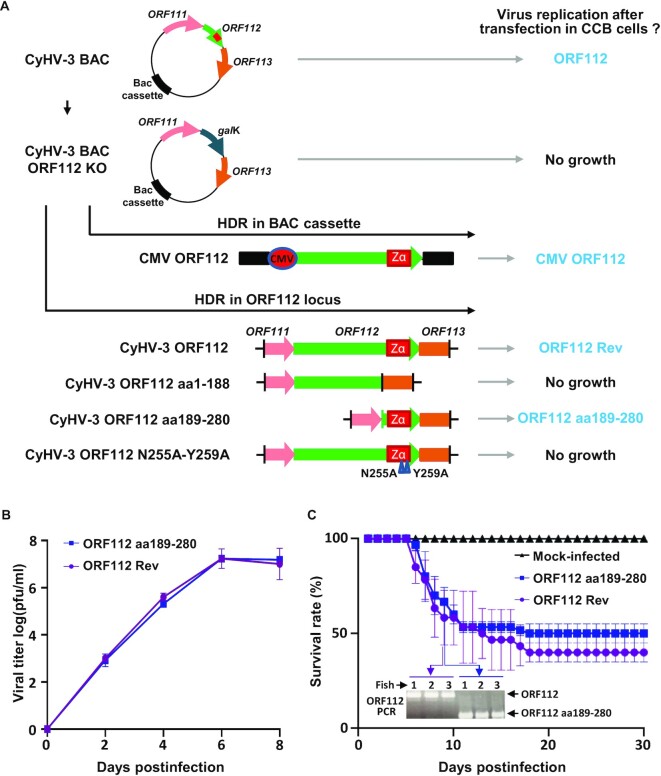
ORF112 Zα domain is essential and sufficient for CyHV-3 growth in cell culture and virulence *in vivo*. (**A**) Flowchart of the production of CyHV-3 ORF112 recombinants. Schematic representation of WT CyHV-3 BAC and derived CyHV-3 BAC ORF112 KO. The ability of different constructions to rescue viral growth after cotransfection with the CyHV-3 BAC ORF112 KO was tested. When viral growth was observed, recombinants were given a short name (in blue) to simplify the reading of the text. (**B**) Viral growth assay of the indicated CyHV-3 recombinants. Data are presented as the means ± the SEM of triplicate measurements. No statistical differences were detected. (**C**) Virulence in carp of the indicated CyHV-3 recombinants. No statistical differences were detected between the two viruses. PCR analyses performed on randomly selected dead fish confirmed the absence of cross-contamination.

The CyHV-3 BAC ORF112 KO recombinant plasmid was produced based on the strategy outlined in Figure 2A by using galactokinase gene (*gal*K) positive selection in bacteria (30,31). *ORF112* (initiation codon to stop codon) was replaced by a *gal*K expression cassette using homologous recombination. The ORF112 Del *gal*K recombination cassette was produced by PCR using the primers (Eurogentec) listed in Supplementary Table S1 and the p*gal*K vector as template. The resulting ORF112 Del *gal*K cassette consisted of the *gal*K gene flanked by 50 bp sequences homologous to the regions of the CyHV-3 genome immediately upstream and downstream of *ORF112*.

### Reconstitution of infectious virus from the CyHV-3 BAC plasmid and the CyHV-3 BAC ORF112 KO plasmid

BAC plasmids were transfected into permissive CCB cells as described previously (27). Transfection of the plasmids and potential reconstitution of infectious virus were monitored by epifluorescence microscopy since the BAC cassette encoded by plasmids and derived viruses contained an EGFP expression cassette. This experiment revealed the inability of the CyHV-3 BAC ORF112 KO plasmid to reconstitute virions (Figure 2A). The possibility of rescuing the infectivity of this plasmid was tested by cotransfecting various recombination cassettes (provided as recombination fragment and not as source of ectopic expression) into eukaryotic cells to induce homologous recombination either in the BAC cassette (Figure 2A) or in the *ORF112* locus (Figures 2A, 6C, 9A, D and F and 10) of the CyHV-3 BAC plasmid. First, a pcDNA3.1 vector (addgene, V790-20) encoding ORF112 under control of the human cytomegalovirus (CMV) immediate-early (IE) promoter was used to target insertion of the pcDNA3.1 ORF112 vector into the BAC cassette (the BAC cassette and the pcDNA3.1 vector share a large homologous region) (Figure 2A). Second, to induce recombination in the *ORF112* locus (Figures 2A, 6C, 9A, D and F and 10), various inserts flanked by 500 bp sequences homologous to the CyHV-3 genome upstream of the ORF112 initiation codon and downstream from the ORF112 stop codon were cloned into the pGEMT vector (Promega, A137A). These inserts encoded Zα domains or dsRNA-binding domains (the amino acid sequences of the proteins tested are provided in Supplementary Table S2). All domain delimitations were performed using the prediction software SMART (32). Further confirmations were done for Zα domains for which the 3D-structure was available in PDBs. Cloning of recombination cassettes was performed in pGEMT vector using the primers listed in Supplementary Table S1. PCR amplicons were assembled using the NEBuilder Assembly kit (New England Biolabs, M5520AA).

### Genetic characterization of CyHV-3 recombinant BAC plasmids and derived recombinant viruses

CyHV-3 BAC recombinant plasmids and derived recombinant viruses were characterized by PCR, by sequencing the regions of interest, and by assessing the sizes of SacI fragments by agarose gel electrophoresis (33). CyHV-3 recombinant viruses ORF112 Rev and ORF112 aa189-280 were characterized further by full-length genome sequencing as described previously (28).

### Multistep growth curves

These assays were performed as described previously (28). Briefly, triplicate cultures of CCB cells were infected with CyHV-3 strains at a multiplicity of infection (MOI) of 0.005 plaque forming unit (PFU)/cell. After incubating for 2 h, the cells were washed with PBS and overlaid with DMEM (Gibco, 31885–023) containing 4.5 g/l glucose and 10% (v/v) fetal calf serum (FCS). The supernatant was removed from the infected cultures at successive intervals (2, 4, 6 and 8 days postinfection), and stored at -80°C. Viral titers were determined by duplicate plaque assays in CCB cells.

### Fish

Conventional common carp (*Cyprinus carpio carpio*) were kept in 60 l tanks at 24°C. Water parameters were checked twice per week. Microbiological, parasitic and clinical examinations were conducted immediately prior to the experiments to ensure that the fish were healthy. Experiments were preceded by an acclimatization period of 2 weeks.

### Infection of fish with CyHV-3

Fish (4 months old, average weight 4.8 g ± 0.95) were inoculated by placing them in water containing the virus (800 PFU/ml of water) for 2 h under constant aeration at 24°C, with the volume of water adjusted to reach a biomass of 10%. Mock-infected fish were treated comparably but in non-infectious water. At the end of the inoculation period, the fish were returned to 60 l tanks. For each condition, triplicate groups of 20 fish were tested. Fish were observed over a period of 1 month. Fish reaching the ending points defined in the bioethics protocol were euthanized. Survival was recorded according to time and expressed as the mean ± SD of the triplicate data.

### Ethics statement

The experiments, the maintenance, and the care of the fish complied with the guidelines of the European Convention for the Protection of Vertebrate Animals used for Experimental and other Scientific Purposes (CETS no. 123). The animal studies were approved by the local ethics committee of the University of Liège, Belgium (laboratory accreditation no. LA1610008, protocol no. 2130). All efforts were made to minimize animal suffering.

### Construction of eukaryotic expression vectors

Recombinant forms of CyHV-3 ORF112 were amplified by PCR (Phusion polymerase, New England Biolabs, M0531L) using the FL BAC plasmid as a template. The primers used (Eurogentec) are listed in Supplementary Table S1. PCR products were cloned by homologous recombination in pEFIN-3 plasmid linearized by BamHI using the NEBuilder Assembly kit (New England Biolabs, M5520AA). All constructs were confirmed by sequencing.

### Production and purification of recombinant proteins using a prokaryotic expression system

Twenty recombinant fluorescent proteins were produced in bacteria. These proteins are described in Supplementary Table S2 (see page related to fluorescent proteins). Coding inserts were amplified by PCR using the primers listed in Supplementary Table S1 and then cloned into the pET.15b vector (Novagen, 69661), resulting in the expression of N-terminal 12xHis tagged fluorescent (GFP or mCherry) recombinant proteins. Proteins were expressed in *Escherichia coli* BL21-pLys cells (Promega, L1195) after induction with 1 mM IPTG (Invitrogen, 15529019) overnight at 20°C. Purification of His-tagged fusion proteins was performed using Ni-NTA beads as described previously (34). Elution of absorbed proteins was performed with 50 mM Tris–Cl (pH 7.9), 500 mM NaCl and 250 mM imidazole. Purified proteins were stored at −80°C in 25% (v/v) glycerol. Protein purity was checked by Coomassie blue staining. Protein concentrations were determined by measuring absorbance at 280 nm (SimpliNano; Biochrom) and by performing a Bradford protein assay using a Micro BCATM protein assay kit (Thermo Fisher Scientific, 23235).

### Cell treatment with arsenite

To induce SG formation, HeLa cells were incubated 24 h posttransfection in culture medium supplemented with 0.5 mM sodium arsenite (Sigma-Aldrich, S7400) for 30 min at 37°C. Immediately after arsenite treatment, cells were treated for indirect immunofluorescent staining of the SG marker HuR using mAb 3A2 as primary antibody (dilution of 1:1000).

### Indirect immunofluorescence staining

With exception of anti-Z-RNA detection (see below) all indirect immunofluorescence staining were performed as follows. Cells grown on glass coverslips were fixed in PBS containing 4% (w/v) paraformaldehyde (PAF) at 4°C for 15 min and then at 20°C for 10 min. After washing with PBS, samples were permeabilized in PBS containing 0.5% (v/v) Triton-X100, 5% (v/v) FCS, and 1% (w/v) BSA at 37°C for 30 min. For immunofluorescent staining, primary antibodies were diluted in the permeabilization buffer and incubated on cells for 1 h at 37°C. Washes were then performed with PBS. Depending to the primary antibody used, Alexa Fluor 568 goat anti-rabbit (Invitrogen, A11011) or 488 goat anti-mouse immunoglobulins (Invitrogen, A32723) were used as secondary antibodies (1:1000 dilution). After washing, cells were mounted by using Vectashield mounting medium containing 4,6-diamidino-2-phenylindole (DAPI) (Vector Laboratories, H-1200).

### Characterization and staining of ribonucleoprotein condensates

CCB cells grown on 12 mm glass coverslips were fixed with 2% (w/v) PAF for 10 min at room temperature followed by an incubation for 20 min at −20°C in −80°C pre-cooled methanol. Cells were further permeabilized in PBS containing 0.5% (v/v) Triton-X100, 5% (v/v) FCS, and 1% (w/v) BSA at 37°C for 30 min. The cells were then submitted to enzymatic treatment with the following enzymes to characterise ribonucleoprotein (RNP) condensates: RNase A (2.5 mg/mL, Thermo Fisher Scientific, EN0531), RNase H, RNase IF, RNase III and DNase I (all at 100 U/ml, New England Biolab, M0297L, M0243L, G6171A and M0303L, respectively). Digestion with RNase A was performed in PBS for 1 h at 37°C. For RNases H, IF and III and for DNase I, digestions were performed for 1 h at 37°C in their respective buffer provided in the kit. Indirect immunofluorescence staining was then performed using the protocol described above with α-dsRNA (1:100 dilution) and α-ORF112 (1:300 dilution) as primary antibodies.

### Indirect immunofluorescence staining of Z-RNA

This protocol was adapted based on previous publications (4,35). Cells grown on glass coverslips were first incubated in −80°C pre-cooled methanol for 10 min at −20°C. After washing with PBS, cells were further fixed in PBS containing 4% (w/v) PAF at 4°C for 15 min and then at 20°C for 10 min. After extensive washing with PBS, cells were permeabilized and treated by proteinase K (0.25 mg/ml Sigma-Aldrich, P2308) in PBS containing 0.25% (v/v) Triton-X100 for 30 min at 37°C to unmask mAb Z22 epitope. After washing with PBS, samples were incubated in PBS containing 0.25% (v/v) Triton-X100, 5% (v/v) FCS, 1% (w/v) BSA and protease inhibitors (cOmplete, Sigma-Aldrich, 11697498001). Immunofluorescent staining and washes were performed in this buffer. When required RNase III digestion was also performed in this buffer before immunostaining.

### Confocal microscopy and image analysis

Samples were analyzed by confocal microscopy using a Leica SP5 confocal microscope and images were compiled using ImageJ Software (36). Leica Application Suite Advanced Fluorescence (LAS-AF) software was used to quantify the relative intensities of fluorochromes. Counting of cells in which ORF112 recombinant forms were recruited into SGs was carried out by quantitative analysis of images by the open-source software CellProfiler (37).

### Fluorescence localization after photoconversion

Fluorescence localization after photoconversion (FLAP) was used to study the localization of Dendra2 fusion proteins expressed by CyHV-3 recombinant strains in living cells according to time. CCB cells were plated in a Cellview microplate 96-well plate (Greiner Bio-one, 627965) and grown overnight. The cells were then infected with recombinant CyHV-3 strains expressing Dendra2 recombinant proteins at a MOI of 1 PFU/cell. Live cell imaging was performed 48 h postinfection using a ZEISS LSM880 (Elyra S1 system equipped with Lattice SIM). The cells were maintained at 5% CO_2_ and 25°C during observation with a 63× objective. Analyses were performed sequentially. First, an argon laser emitting at 488 nm wavelength (1% intensity, fast Airyscan mode) was used to detect green fluorescence. After identifying a region of interest (ROI), conversion was induced by the exposure of the ROI to 405 nm excitation light (the power of the laser was adjusted to 70% of its maximum). Fifty iterations were applied, and the conversion was repeated every minute during the acquisition period. Next, images were taken every 30 s for 15 min by using a neon laser emitting at 561 nm (2% intensity and fast Airyscan mode). To avoid phototoxicity to the cells, the gain was fixed at 800. For image analyses, a two-step approach was adopted. First, fast Airyscan processing was applied by ZENblack software, and then final processing was performed using Imaris 9.5 software.

### Induction of liquid–liquid phase separation

The ability of purified proteins to induce LLPS was tested as follows. Samples were prepared by mixing purified proteins (final concentration of 10 μM) with 5% (v/v) PEG-6000 (Sigma-Aldrich, 807491) or Z-RNA (CG-repeat of 6-mer RNA duplex, (C**m^8^Gm**C**m^8^Gm**CG), m^8^Gm-modified are presented in bold) at a final concentration of 10 μM or FAM-labelled Z-RNA (FAM-Z-RNA) (CG-repeat of 6-mer RNA duplex, FAM-r(C**m^8^Gm**C**m^8^Gm**CG), m^8^Gm-modified are presented in bold) at a final concentration of 10 μM (38,39). Ten μl of this mixture was then loaded into cell-counting slides (Bio-Rad, 1450015). Sealed chambers containing mixture of proteins and PEG were incubated for 1 h at room temperature. For Z-RNA-induced LLPS, the slides were briefly cooled on ice and incubated overnight at 4°C. For imaging, droplets were observed on a slide using a LSM 510 Meta confocal laser scanning system (TCS SP5; Leica). Fluorescence images of the droplets were taken as a series of confocal z-stacks and displayed as the maximum intensity projection by ImageJ. LLPS quantification ImageJ and an in-house written macro (Supplementary Table S3) were used for LLPS quantification. Image thresholding was made by Huang's method (40). The ‘white objects on black background’ option was used, and ‘set measurement’ and ‘analyze particles’ were run.

### Fluorescence recovery after photobleaching

Fluorescence recovery after photobleaching (FRAP) was studied in liquid droplets containing mCherry-tagged proteins by confocal microscopy (LSM 880 Meta plus Zeiss Axiovert zoom, Zeiss) at room temperature. ROI was first photobleached by excitation at 405 nm. The fluorescence intensity of the photobleached area was then collected every 2 s. Image intensity was measured by Mean ROI in the Zeiss Zen software during the acquisition time, and graphs drawn by Prism8 (GraphPad).

### Statistical analysis

Viral growth data were compared using two-way ANOVA with interactions followed by the post-hoc t-test using GraphPad Prism 8. Survival results after inoculation of fish were compared by GraphPad Prism 8 using the Log-rank (Mantel-Cox) test and the Gehan–Breslow–Wilcoxon test (41). The percentages of cells in which ORF112 recombinant forms colocalized with SGs were compared by unpaired t-test using GraphPad Prism8 software. Independently of the test, levels of statistical significance were set at *P* < 0.05, *P* < 0.01 or *P* < 0.001.

## RESULTS

### The ORF112 protein is highly conserved among CyHV-3 strains and consists of an N-terminal intrinsically disordered region and a C-terminal Zα domain

In earlier studies, we showed using the FL strain of CyHV-3 that the *ORF112* gene leads to the expression of a protein (ORF112) both in infected cells (24) and in virions (42,43). The nucleotide sequence of *ORF112* encoded by seven strains representative of the CyHV-3 viral species revealed that, with the exception of a deletion of two codons at the beginning of the protein in two strains, all strains expressed an identical sequence (28). *In silico* analyses of ORF112 encoded by most strains revealed a 280 aa protein consisting of a large N-terminal region encoding an intrinsically disordered region (IDR) and a C-terminal Zα domain (Figure 1B). Immunostaining of infected cells indicated that all strains expressed ORF112 (Figure 1C). Western blot analyses confirmed this conclusion and showed that the *ORF112* gene leads to the expression of a single protein of ∼30 kDa, corresponding to the expected molecular weight of the ORF112 protein (Figure 1D). These results suggested that the ORF112 protein containing a Zα domain is highly conserved among CyHV-3 strains and therefore is likely to play important roles during viral replication.

### ORF112 is essential for CyHV-3 growth in cell culture

The roles of ORF112 and its domains in viral replication in cell culture were investigated using BAC cloning to generate viral mutants (Figure 2A). To facilitate the reconstitution of infectious viruses from recombinant plasmids, the BAC cassette was left in the viral genome, leading to viruses expressing a truncated form of CyHV-3 thymidine kinase (TK, encoded by *ORF55*) and enhanced green fluorescent protein (EGFP, the BAC cassette was inserted at the 3’ end of ORF55 and encoded an EGFP expression cassette). TK truncation is known to have no effect on viral growth in cell culture and to induce a mild attenuation *in vivo* (27).

A recombinant BAC plasmid lacking *ORF112* was produced by replacing *ORF112* with a *gal*K expression cassette (Figure 2A, CyHV-3 BAC ORF112 KO plasmid). The ability of this recombinant plasmid to reconstitute infectious virus after transfection into permissive CCB cells was investigated. Examination of cell cultures at 6 days posttransfection revealed the formation of viral plaques for the CyHV-3 BAC plasmid (used as a positive control) but not for the CyHV-3 BAC ORF112 KO, suggesting that ORF112 is essential for viral growth in cell culture. To exclude the possibility that the lack of infectivity of the ORF112 KO plasmid resulted from unexpected mutations generated during BAC manipulation, this plasmid was transfected into CCB cells together with a wild-type (WT) DNA fragment containing *ORF112* and flanking regions to induce reversion to a WT *ORF112* locus after homologous recombination in eukaryotic cells. This led to viral growth and recovery of the revertant strain (ORF112 Rev, Figure 2A), thereby demonstrating that the lack of infectivity of the ORF112 KO plasmid was due directly or indirectly to the absence of *ORF112*. To exclude the possibility that the lack of infectivity observed for the ORF112 KO plasmid resulted from an indirect effect of the ORF112 deletion on the expression of a nearby essential gene (polar effect), attempts were made to rescue viral growth by inserting an ORF112 expression cassette elsewhere in the genome. The ORF112 KO plasmid was transfected into CCB cells together with a pcDNA vector encoding an ORF112 expression cassette under the control of CMV IE promoter. The expression cassette was flanked by homologous sequences to the BAC cassette to target its insertion by homologous recombination. This approach led to viral growth and the production of the CMV ORF112 strain (Figure 2A), thereby demonstrating that the lack of infectivity of the ORF112 KO plasmid was not due to a polar effect caused by the lack of *ORF112*.

These results strongly suggested that ORF112 is essential for CyHV-3 growth in cell culture. However, the approach used relied on an artificial mode of inoculation involving the transfection into permissive cells of naked DNA consisting of the viral genome cloned as a BAC. To exclude the possibility that ORF112 is essential for reconstituting infectious virions under these artificial conditions, while not being essential for the replication of reconstituted virus, the requirement of ORF112 for viral growth in cell culture was examined using a coinfection-based approach involving a helper virus (Supplementary Figure S1). This approach confirmed that ORF112 is essential for CyHV-3 growth in cell culture.

### ORF112 Zα domain is essential and sufficient for CyHV-3 growth in cell culture and virulence *in vivo*

To identify whether both domains of ORF112 contribute to the requirement of ORF112 for viral growth in cell culture, the CyHV-3 BAC ORF112 KO plasmid was transfected into CCB cells together with a DNA fragment encoding part of ORF112 (aa1–188 or aa189–280) and flanking regions, to induce homologous recombination in the *ORF112* locus of the CyHV-3 genome (Figure 2A). CyHV-3 genome encoding a truncation of ORF112 to the N-terminal domain was not able to produce infectious particles (Figure 2A, CyHV-3 ORF112 aa1–188 plasmid). In contrast, recombinant CyHV-3 genome encoding the C-terminal domain of ORF112 induced viral replication (Figure 2A, CyHV-3 ORF112 aa189–280 plasmid). The complete genome sequence of the resulting virus (ORF112 aa189–280) was verified and shown to lack additional mutations. These results suggested that the N-terminal domain of ORF112 is not essential for viral growth in cell culture but that the C-terminal Zα domain is essential and sufficient.

The data described above suggested that ORF112 is essential for viral growth because of the interactions of its Zα domain with nucleic acids in Z-conformation. Previous crystallographic studies have identified the importance of two critical residues (N255 and Y259 of CyHV-3 ORF112) in the Zα domains for such interactions (24,44). The impact of site-directed mutagenesis of these residues (N255A, Y259A) was tested on the ability of CyHV-3 to grow in cell culture. The CyHV-3 BAC ORF112 KO plasmid was transfected into CCB cells together with a DNA fragment containing mutated ORF112 sequences (N255A and Y259A) and flanking regions to facilitate homologous recombination (Figure 2A). The lethal effect observed for the N255A-Y259A mutation supports a key role for ORF112 as a Z-nucleic acid-binding protein in the replication cycle of CyHV-3.

The above data indicated that the N-terminal domain of ORF112 containing an IDR is dispensable for CyHV-3 growth in cell culture. In contrast, the C-terminal Zα domain is essential and sufficient for efficient growth. Next, to determine a potential quantitative impact of the ORF112 N-terminal domain on CyHV-3 replication in cell culture and virulence *in vivo*, we compared the ORF112 Rev (encoding WT ORF112) and the ORF112 aa189–280 (encoding only the C-terminal Zα domain) recombinant viruses. Viral growth in cell culture was investigated by multistep growth assays (Figure 2B). The results suggested no significant differences between the two recombinants. Similarly, inoculation of common carp by the two recombinants revealed comparable levels of virulence (Figure 2C). PCR characterization of randomly selected infected fish supported the absence of cross-contamination between the groups.

These results suggested that the expression of only the Zα domain of ORF112 was sufficient for normal virus replication in cell culture and virulence in carp. In contrast, its deletion was lethal for the virus. Unexpectedly, these observations revealed the potential of the CyHV-3 model as a unique platform to study in living cells the properties of Zα domains expressed alone.

### ORF112 and the ORF112 Zα domain accumulate in dynamic ribonucleoprotein condensates in CyHV-3 infected cells

**Figure 3.**
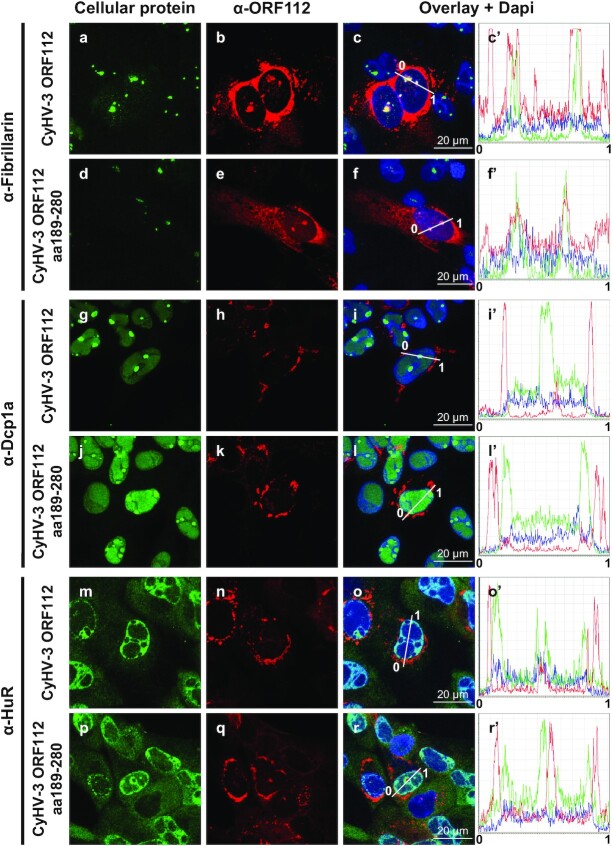
Subcellular localization of ORF112 and ORF112 Zα domain in CyHV-3 infected cells. Cells infected with CyHV-3 recombinants expressing ORF112 (full-length protein, CyHV-3 ORF112) or ORF112 aa189-280 (Zα domain only, CyHV-3 ORF112 aa189–280) were fixed, permeabilized, and treated for double immunofluorescence staining of the indicated cellular protein (green signal) and ORF112 (α-ORF112, red signal). DNA was stained with Dapi (blue signal). Line intensity profiles are shown in the right panels.

The results above suggested that the N-terminal domain of ORF112 containing an IDR has no essential biological functions or that its functions are redundant to those of the ORF112 Zα domain. To compare further the full-length ORF112 protein and the truncated form restricted to the Zα domain, we investigated subcellular localization during viral infection (Figure 3). Both forms expressed comparable subcellular distribution, with the vast majority of the protein concentrated in cytosolic condensates around the nucleus. Both proteins were also expressed at much lower levels in nucleoli (colocalization with fibrillarin, Figure 3, panels a–f’). This subcellular distribution is consistent with our earlier observations (see Figure 7 of (24)). Interestingly, ORF112 did not colocalised with markers of P-bodies (Figure 3, α-Dcp1a, panels g and l’) or of stress granules (Figure 3, α-HuR, panels m and r’).

**Figure 4.**
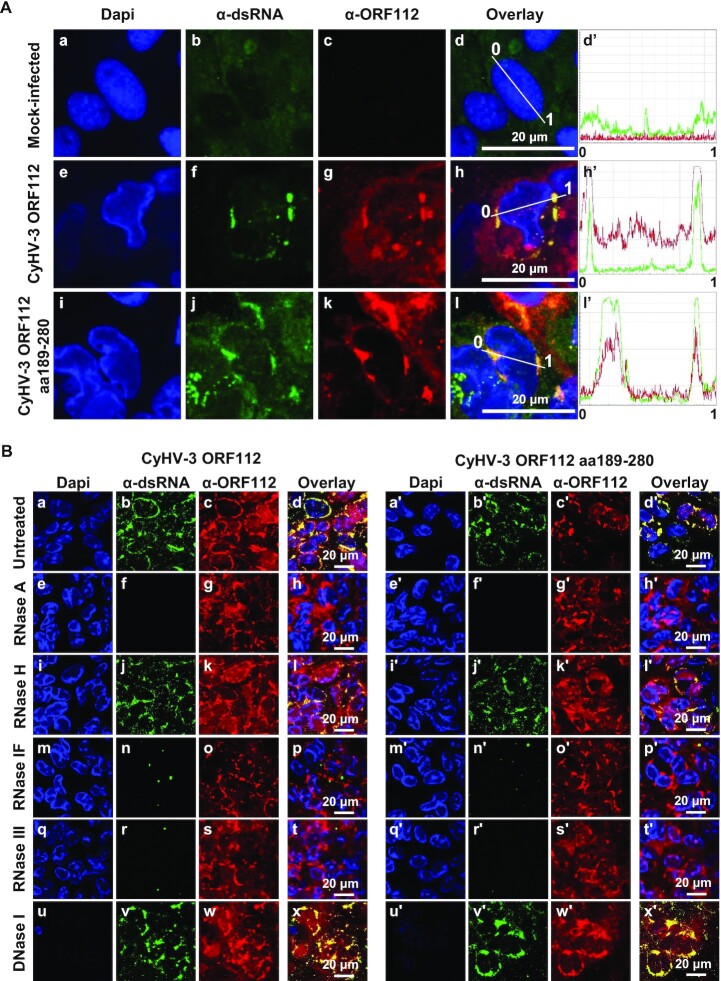
ORF112 and ORF112 Zα domain accumulate in RNP condensates in infected cells. (**A**) Cells infected with CyHV-3 recombinants expressing ORF112 (full-length protein, CyHV-3 ORF112) or ORF112 aa189–280 (Zα domain only, CyHV-3 ORF112 aa189–280) were fixed, permeabilized, and treated for double immunofluorescence staining of dsRNA (α-dsRNA, green signal) and ORF112 (α-ORF112, red signal). DNA was stained with Dapi (blue signal). Line intensity profiles are shown in the right panels. (**B**) Cells infected with CyHV-3 recombinants expressing ORF112 (full-length protein, left panels) or ORF112 aa189–280 (Zα domain only, right panels) were fixed, permeabilized and treated with the indicated enzymes before immunostaining of dsRNA (α-dsRNA, green signal) and ORF112 (α-ORF112, red signal). DNA was stained with Dapi (blue signal).

Recent studies suggested that Zα domains interact mainly with Z-RNA in the context of viral infection (4–6). Moreover, herpesvirus infections have been reported to be associated with the expression of cytosolic dsRNA (45). For these reasons, we investigated whether the ORF112 cytosolic granules represent RNPs. Immunostaining of infected cells with α-ORF112 antibodies and α-dsRNA mAb J2 revealed that both the full-length ORF112 and ORF112 Zα colocalized with dsRNA, supporting the hypothesis above (Figure 4A, panels e–h’, and i–l’). To control the specificity of staining by mAb J2, fixed and permeabilized cells were submitted to enzymatic treatments before immunostaining (Figure 4B). Treatment with RNase A (cleavage of ssRNA and dsRNA as well as the RNA strand in the RNA-DNA hybrids) (panels e–h and e’–h’), RNase IF (cleavage of ssRNA and dsRNA) (panels m–p and m’–p’), and RNase III (cleavage restricted to dsRNA) (panels q–t and q’-t’) erased mAb J2 staining in RNP condensates. In contrast, RNase H (cleaving the RNA strand in an RNA-DNA heteroduplex) (panels i–l and i’–l’) and DNase I (cleaving dsDNA) (panels u–x and u’–x’) did not significantly affect mAb J2 staining. Together, these data indicated that the RNP condensates containing ORF112 or ORF112 Zα also contain dsRNA.

**Figure 5.**
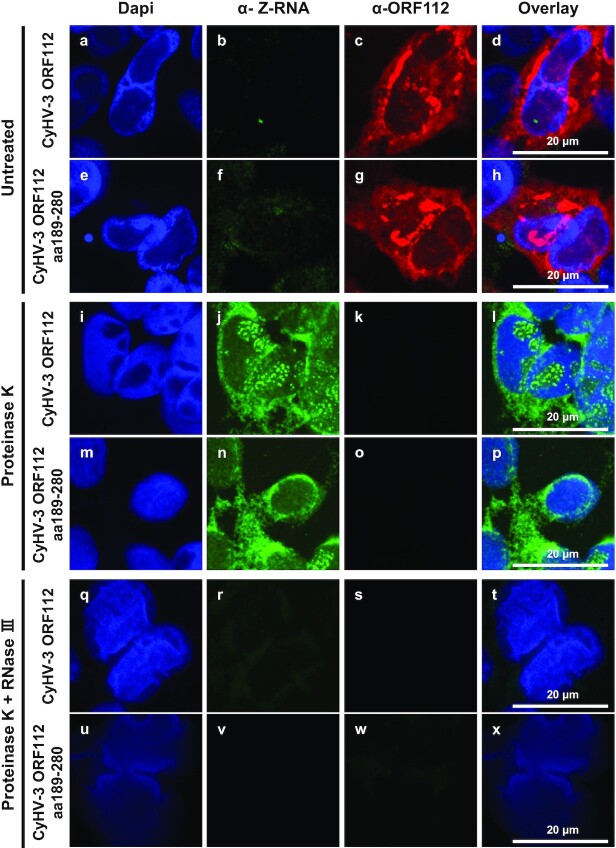
CyHV-3 infected cells contain Z-RNA. Cells infected with CyHV-3 recombinants expressing ORF112 (full-length protein, CyHV-3 ORF112) or ORF112 aa189-280 (Zα domain only, CyHV-3 ORF112 aa189–280) were fixed and permeabilized. Cells were then mock-treated (untreated, two upper rows of panel) or treated with Proteinase K (two middle rows of panel) or treated with both Proteinase K and RNase III (two lower rows of panel) before double immunofluorescence staining of Z-RNA (α-Z-RNA, green signal) and ORF112 (α-ORF112, red signal). DNA was stained with Dapi (blue signal).

The colocalization observed between ORF112 and dsRNA in CyHV-3 infected cells together with the ability of ORF112 to bind to Z-DNA (24) suggest that CyHV-3 infected cells express Z-RNA. To test this hypothesis, cells infected by CyHV-3 (expressing either ORF112 or only ORF112 Zα domain) were subjected to double immunofluorescent staining using mAb Z22 (4,35) raised against Z-RNA/DNA and polyclonal antibodies against ORF112 (Figure 5). Immunostaining of cells untreated with proteinase K revealed the usual pattern of ORF112 but nearly no staining by mAb Z22 (Figure 5, panels a–h). Cells treated with proteinase K before immunostaining revealed the opposite results (Figure 5 panels i-p). Interestingly, mAb Z22 revealed a pattern similar to the one observed for ORF112 in untreated cells with exception that additional multiple positive foci were observed in the nucleoplasm (where viral DNA replication takes place) in a fraction of the infected cells. No more staining was observed with mAb Z22 when cells were treated with proteinase K and RNase III before staining (Figure 5, panels q–x). These results showed that ORF112 outcompetes the binding of mAb Z22 to its epitope thereby supporting the conclusion that this viral protein binds to Z-RNA in infected cells.

**Figure 6.**
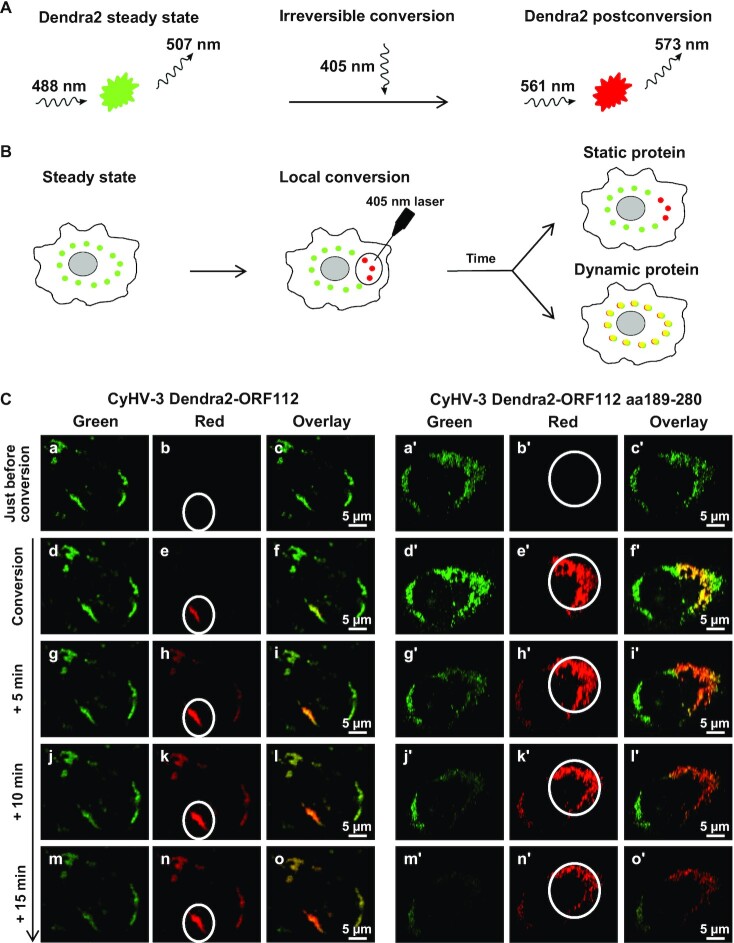
ORF112 and ORF112 Zα domain accumulate in dynamic RNP condensates in infected cells. (**A**) Photoconversion of Dendra2 by illumination at 405 nm. (**B**) Schematic representation of photoconversion in living cells to determine whether a protein is static or dynamic. (**C**) Living cells infected with CyHV-3 recombinants expressing Dendra2 N-terminal tagged ORF112 (full length protein, left panels) or Dendra2 N-terminal tagged ORF112 aa189-280 (Zα domain only, right panels) were submitted to green-to-red photoconversion on the indicated ROI (FLAP experiment) every 5 min. Representative pictures collected according to time postinitiation of the conversion are presented.

Next, we investigated using photoconversion in living cells whether the RNP condensates containing ORF112 or ORF112 Zα in CyHV-3 infected cells were static or dynamic (Figure 6A and B). To address this question, two new CyHV-3 recombinant strains were produced. These strains expressed ORF112, or ORF112 Zα fused to the C-terminus of the photoconvertible Dendra2 fluorescent protein (Figure 6A) (46). The fusion of Dendra2 to ORF112 or ORF112 Zα did not affect the essential functions of these proteins as supported by the ability of the recombinant viruses to replicate in cells. The similar subcellular distribution observed for ORF112 tagged, and untagged recombinant proteins further supported the absence of negative effect of the tag on the ORF112 Zα proteins (compare the results of Figures 3 and 6C). Living cells infected with the CyHV-3 Dendra2-ORF112 strain and the CyHV-3 Dendra2-ORF112 Zα strain were observed by confocal microscopy 48 h after inoculation (Figure 6C). Similar observations were made for the two forms of the protein. Analysis of the cells before conversion revealed the distribution of the fusion protein only in the green channel (Figure 6C, first line). A ROI encompassing an RNP was then selected and submitted to green-to-red conversion by UV (405 nm) excitation, and led to the appearance of a red signal in the ROI (Figure 6C, second line). At increasing times postconversion, the red signal appeared in RNPs distant from the converted ROI, suggesting an intense trafficking of ORF112 and ORF112 Zα, and the dynamic nature of the RNPs (Figure 6B and C).

### ORF112 is recruited in stress granules through different mechanisms

**Figure 7.**
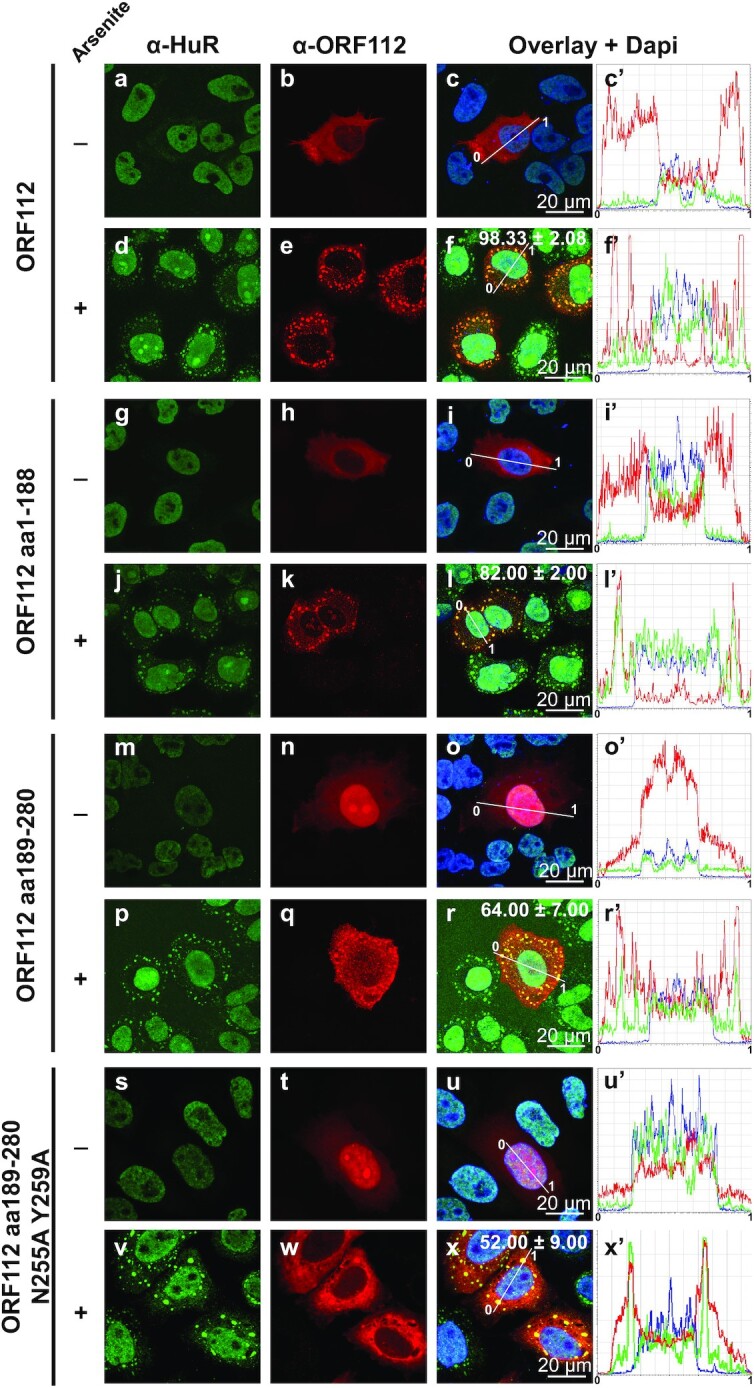
ORF112 and its two subdomains colocalize with arsenite-induced stress granules. HeLa cells transfected with plasmids encoding the indicated proteins were mock-treated (–) or treated with arsenite, then processed for immunostaining of HuR and ORF112. The numbers in panels f, l, r and x refer to the percentage of cells, in which ORF112 was observed colocalizing with SGs (means ± the SEM of triplicate analyses of 100 randomly selected transfected cells).

RNP granule assembly and maintenance mechanisms are not yet fully understood (47,48). Stress granules represent one of the prominent types of RNP granules. They are dynamic and reversible cytoplasmic assemblies formed through LLPS in eukaryotic cells, in response to various stresses. We showed previously that ORF112 colocalized with arsenite-induced SGs in CyHV-3 infected cells (24). This was interpreted as being due to the binding of ORF112 through its Zα domain to Z-RNA expressed in SGs. Here, we revisited this hypothesis in the context of ectopic expression (Figure 7). Full-length ORF112, the N-terminal domain (aa1–188) of ORF112, the WT ORF112 Zα domain (aa189–280), or the mutated ORF112 Zα domain (aa189–280 N255A-Y259A) were expressed transiently in HeLa cells. Regardless of the expression construct tested, transfected control cells untreated with arsenite did not exhibit SG formation, as revealed by the subcellular localization of HuR in the nucleoplasm (Figure 7, panels a–c’, g–i', m–o’, and s–u’). Upon arsenite treatment, nearly all cells expressed SGs, as revealed by the appearance of HuR positive granules in the cytosol. Regardless of the ORF112 construct tested, the protein relocalized into SGs. However, the proportion of cells expressing perfect colocalization between HuR granules and the ORF112 recombinant proteins varied according to the construct tested. Thus, the highest proportion was observed for full-length ORF112 (98%), followed in decreasing order by the N-terminal domain (82%), the Zα domain (64%) and the mutated Zα domain with deficient Z-binding activity (52%). Statistical analyses of these data revealed that, with the exception of the last two scores that were not detected as significantly different (*P* = 0.142), comparison of all other pairs led to significant differences (*P* < 0.05 or lower). These data suggested that relocalization of ORF112 in SGs results from interactions involving both the N-terminal domain containing an IDR and the C-terminal Zα domain. Moreover, the ability (though reduced) of the mutated ORF112 Zα domain with deficient Z-binding activity to relocalize into SGs (Figure 7, panel x) suggested that the WT ORF112 Zα domain relocalized in SGs on the basis of two different mechanisms, respectively dependent on and independent of Z-binding activity.

Stress granules are membrane-less organelles (reviewed in (49,50)) formed through LLPS. Our computational analyses predicted that the N-terminal region of ORF112 contains an IDR encoding at least two low-complexity domains (LCDs), one Q-rich and the other H-rich. Both Q-rich and H-rich LCDs have been shown to drive LLPS (51,52). The ORF112 Zα domain is more positively charged than other Zα domains. In addition to LLPS induced by LCDs, it has been also shown that weak interactions between positively charged peptides and nucleic acids promote the formation of the core of RNPs through LLPS (53–55). This information led us to hypothesize that both the N-terminal domain and the C-terminal Zα domain of ORF112 can induce LLPS. According to this hypothesis, the ability of CyHV-3 to replicate without the N-terminal domain of ORF112 would be due to the redundant ability of both the N-terminal domain and the C-terminal Zα domain to form LLPS.

### Both the ORF112 N-terminal domain and ORF112 C-terminal Zα domain can perform LLPS

**Figure 8.**
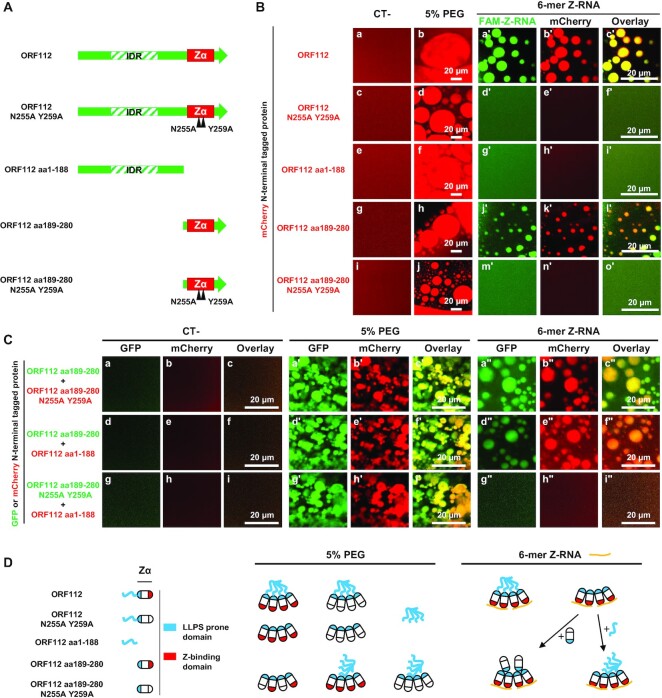
Ability of ORF112 and its subdomains to mediate LLPS induced by PEG or Z-RNA. (**A**) Schematic representation of ORF112 recombinant forms tested. Black triangles illustrate mutation impairing Z-binding activity. (**B**) *In vitro* LLPS induced by incubation of the indicated mCherry N-terminal tagged recombinant forms of ORF112 (10 μM) with PEG-6000 (5%, v/v) (second column) or 10 μM of FAM-Z-RNA (three right columns). See also Supplementary Figures S2 and S3. (**C**) *In vitro* LLPS induced by incubation of the indicated mixture of mCherry (red) and GFP (green) N-terminal tagged recombinant forms of ORF112 (10 μM of each protein) with PEG-6000 (5%, v/v, three middle panels) or 10 μM of Z-RNA (three right panels). (**D**) Schematic representation of the hypothetical interactions leading to the LLPS results observed.

To test the ability of the ORF112 N-terminal domain and the ORF112 C-terminal Zα domain to perform LLPS (Figure 8), we designed and expressed five N-terminal mCherry-tagged proteins corresponding to the full-length ORF112 (ORF112), the full-length ORF112 with mutations impairing Z-binding (ORF112 N255A Y259A), the ORF112 N-terminal domain (ORF112 aa1–188), the ORF112 C-terminal Zα domain (ORF112 aa189–280), and the ORF112 C-terminal Zα domain with mutations impairing Z-binding (ORF112 aa189–280 N255A Y259A) (Figure 8A). LLPS was then induced by adding the molecular crowding agent PEG to purified recombinant proteins. No droplet formation was observed when purified mCherry or purified GFP used as control in the experiment presented in Figure 8B and C were submitted to the test (Supplementary Figure S2A, induction by PEG and by Z-RNA). The formation of droplets was observed comparably for all ORF112 recombinant proteins tested after induction (Figure 8B, second column) (see also results of Figure S3B obtained with untagged ORF112 Zα domain). Droplets then tended over time to fuse together into larger droplets, reflecting their liquid properties. Consequently, the number and size of droplets decreased and increased, respectively, over time (as illustrated in movies S1 and S2 for mCherry N-terminal tagged ORF112). Late after induction, droplets accumulated into large irregular networks exhibiting a hydrogel aspect. To further compare the induction of LLPS observed with the ORF112, ORF122 aa1–188, and ORF112 aa189–280 proteins, we quantified the number, average size, and circularity of droplets according to time postinduction (Supplementary Figure S3A). These analyses suggested that the three proteins tested performed LLPS comparably. LLPS is characterized by a dynamic exchange of molecules between the droplet and the bulk phase (56,57). To characterize further the liquid nature of the droplets observed with ORF112 recombinant proteins, we assessed the mobility of the molecules between the droplet and the bulk phase using FRAP. This approach consisted of quantifying over time the recovery of fluorescence of a single droplet after its photobleaching (Supplementary Figure S3B). These data showed a rapid recovery of fluorescence after bleaching for all three tested recombinant forms of ORF112, thus demonstrating the highly dynamic exchange of molecules between the droplets and the surrounding solution even after 1 h of induction (Supplementary Figure S3B).

Next, we investigated the ability of the five recombinant forms of ORF112 described above to perform LLPS in the presence of Z-RNA (Figure 8B, right three columns). LLPS was observed only for recombinant proteins encoding a functional Z-binding activity (ORF112, panels a’–c’; ORF112 aa189–280, panels j’–l’), thus demonstrating that this activity is essential for Z-RNA dependent assembly of RNPs by full-length ORF112 and the ORF112 Zα domain. These results, together with the PEG-induced LLPS data described above, suggested that the formation of droplets results from two types of interaction: first, the interaction between the Zα domain and nucleic acids through Z-binding activity leading to nucleation, and second, protein-protein interactions leading to LLPS (Figure 8D). According to this two-step model, proteins unable to perform the first step could be recruited into LLPS droplets by the second type of interaction. To test this hypothesis, we studied the formation of LLPS by a mixture of two different proteins identified based on their fusion with mCherry or GFP fluorescent protein (Figure 8C). LLPS was induced with PEG or Z-RNA. This experiment generated various observations supporting the model described above (schematically represented in Figure 8D). First, the mixture of the ORF112 Zα domain (with Z-binding activity) and the mutated ORF112 Zα domain with impaired Z-binding activity led to the formation of droplets containing both proteins (Figure 8C, panels a’–c’ and a’’–c’’) with both inducers of LLPS. This result indicated the existence of homotypic protein-protein interactions between ORF112 Zα domain molecules that occurred independently of the Z-binding activity. This conclusion is consistent with earlier crystallographic observations of ORF112 Zα domain (12). Second, the mixture of the ORF112 Zα domain with the ORF112 N-terminal domain also led to the formation of droplets containing both proteins (Figure 8C, panels d’–f’ and d’’–f’’) with both inducers of LLPS. This result indicated the existence of heterotypic protein-protein interactions between the ORF112 Zα domain and the ORF112 N-terminal domain molecules. Third, the mixture of the mutated ORF112 Zα domain with impaired Z-binding activity with the ORF112 N-terminal domain led to the formation of droplets containing both proteins (Figure 8C, panels g’–i’ and g’’–i’’) after LLPS induction by PEG but not Z-RNA. These observations further support the existence of heterotypic protein-protein interactions between the Zα domain and the N-terminal domain of ORF112 molecules. These data also confirm that the Z-binding activity is essential for initiating the formation of droplets by the ORF112 Zα domain after induction with Z-RNA.

Taken together, the data above demonstrate that the ORF112 Zα domain can induce LLPS after induction by PEG and Z-RNA. This study is the first to report such activity for a Zα domain. However, it was not possible to determine from these data whether the induction of LLPS by the ORF112 Zα domain is essential for viral replication or whether the ability to induce LLPS is a common feature of Zα domains. Therefore, we investigated these questions further by exploiting the CyHV-3 model as a unique platform for comparing in living cells the exchangeability of Zα domains expressed alone.

### Only certain Zα domains expressed alone are able to rescue the deletion of ORF112

**Figure 9.**
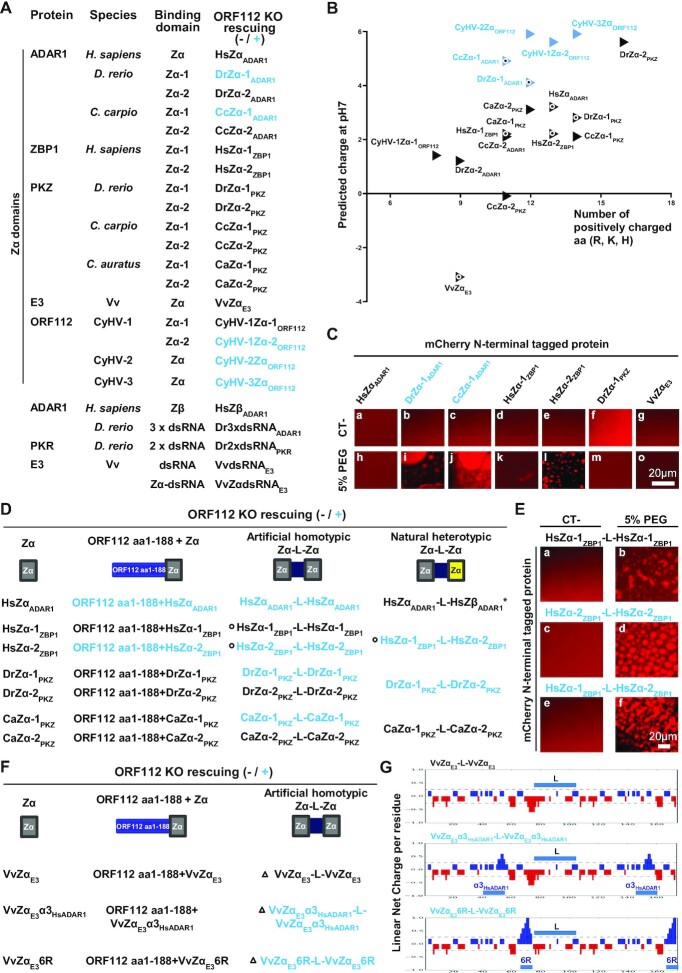
Rescuing of CyHV-3 ORF112 KO requires the expression of a Zα domain-containing protein able to mediate both LLPS and B-to-Z transition. (**A**, **D** and **F**) The ability of Zα and dsRNA binding domains from various origins to rescue the lethal effect of ORF112 deletion on viral growth in cell culture was tested by transfection of the CyHV-3 BAC ORF112 KO plasmid into CCB cells together with a recombining fragment encoding the protein to be tested and *ORF112* flanking regions, in order to induce homologous recombination at the *ORF112* locus (sequences of the proteins tested are provided in Supplementary Table S2). At 6 days posttransfection, the cells were examined by epifluorescence microscopy (the BAC cassette encodes EGFP). Throughout this figure sequences able or not able to rescue virus replication are presented in blue or black, respectively. (**B**) Representation of global predicted charge *versus* the number of positively charged amino acids for the Zα domains listed in panel A. Black circles show the proteins selected for LLPS experiment presented in panel C (a version of this figure including Zα domains of mouse proteins is provided as supplemental material, Figure S4). (**C**) *In vitro* LLPS induced by incubation of the indicated mCherry N-terminal tagged Zα domains (10 μM) with PEG-6000 (5%, v/v). (**D**) Ability of selected Zα domains (left column) to rescue the lethal effect of ORF112 deletion on viral growth in cell culture when expressed in fusion to the N-terminal domain of CyHV-3 ORF112 (ORF112 aa1–188, second column), as artificial homotypic tandem Zα domains joined by a linker (third column) or as natural heterotypic tandem Zα domains (fourth column). The asterisk highlights that HsZβ_ADAR1_ does not have Z-DNA binding activity. Linker sequences were derived from the sequence of the protein from which the first Zα domain was derived. Black circles show the proteins selected for the LLPS experiment presented in panel E and performed as described in panel C. (**F**) Ability of VvZα_E3_ and derived mutant Zα domains (left column) to rescue the lethal effect of ORF112 deletion on viral growth in cell culture when expressed in fusion to the N-terminal domain of CyHV-3 ORF112 (ORF112 aa1–188, second column), and as artificial homotypic tandem Zα domains joined by a linker (sequence of the linker was derived from Hs ZBP1, third column). Black triangles show the proteins selected for net charge distribution analysis presented in panel **G**.

The possibility of rescuing the CyHV-3 ORF112 deletion by Zα domains expressed alone or by other nucleic acid-binding domains (Zβ, dsRNA binding) (Figure 9A) was tested using the approach described in Figure 2A. The CyHV-3 BAC ORF112 KO plasmid was cotransfected into CCB cells together with a recombining fragment encoding the protein to be tested and ORF112 flanking regions to induce homologous directed recombination in the *ORF112* locus. The rescue of CyHV-3 growth was monitored 1 week posttransfection. Only certain Zα domains were able to rescue the lethal effect of ORF112 deletion, these are presented in blue in the right column of Figure 9A. This limited group contained the Zα domains of CyHV-1 (Zα-2) and CyHV-2 (Zα) and the Zα-1 of ADAR1 of cypriniform fishes (*D. rerio* and *C. carpio*). None of the other nucleic acid binding domains tested were able to rescue CyHV-3 growth (Figure 9A, last 5 lines). Of note, this list also contained the E3 protein of Vv (Figure 9A, last line).

### Zα domains expressed alone that can rescue the deletion of CyHV-3 ORF112 have a high positive charge and can mediate LLPS

The experiment described above supported the functional diversity of Zα domains. It suggested the hypothesis that only certain Zα domains can perform LLPS and that this property is essential for CyHV-3 replication. Weak interactions between positively charged peptides and nucleic acid have been shown to promote the formation of the core of RNPs through LLPS (53–55). The relatively high positive charge of the ORF112 Zα domain in comparison to other Zα domains might thus be responsible for the ability of this domain to perform LLPS. Therefore, we investigated whether a correlation exists between the predicted positive charge of Zα domains and their ability to rescue the ORF112 deletion (Figure 9B). Remarkably, the five Zα domains (including the ORF112 Zα domain of CyHV-3) that were able to rescue the ORF112 deletion (Figure 9B, blue triangles) were among the top six most positively charged Zα domains. To test the hypothesis further, seven Zα domains (two able to rescue the ORF112 deletion and five unable to rescue the deletion, Figure 9B, white circle) were selected across the range of predicted charge. These Zα domains were expressed as mCherry N-terminal tagged proteins, purified, and tested for their ability to undergo LLPS (Figure 9C). Consistent with our hypothesis, the two Zα domains that were able to rescue the ORF112 deletion induced LLPS, whereas the other Zα domains that were unable to rescue the deletion induced LLPS at a very low level (HsZα-2_ZBP1_) or not at all.

These results indicated the ability of certain Zα domains to induce LLPS. Consequently, this study led to the discovery of a new property of Zα domains and a new level of functional diversity among Zα domains. It also suggested that CyHV-3 replication requires the expression of a protein capable of both Z-binding activity and LLPS induction. According to this hypothesis, the failure of certain Zα domains to rescue the CyHV-3 ORF112 deletion is due to their inability to induce LLPS. This extended hypothesis was tested using a rational protein engineering strategy as described below.

### CyHV-3 replication requires the expression of a protein that combines three properties: Z-binding activity, LLPS induction, and A-to-Z conversion

The results presented in Figure 8B revealed that the N-terminal domain of CyHV-3 ORF112 containing an IDR is able to induce LLPS. We used this observation to engineer fusion proteins that were expected to combine LLPS induction and Z-binding properties. The CyHV-3 ORF112 N-terminal domain which was unable to rescue the ORF112 deletion and expressed no Z-binding activity, was fused to seven individual Zα domains that were unable to rescue the ORF112 deletion when expressed alone (Figure 9D, left column). The ability of these fusion proteins to rescue the ORF112 deletion was then tested using the approach described above (Figure 2A). The data presented in Figure 9D showed that only two out of the Zα domains gained the ability to rescue the ORF112 deletion when fused to the LLPS-prone ORF112 N-terminal domain. This low rate of gain of function suggested that the protein required to rescue the ORF112 deletion has an additional property lacking from most of the fusion proteins tested. The observation that the two Zα domains that gained the ability to rescue the ORF112 deletion are both good B-to-Z converters (11,17) suggested that this additional property, in addition to Z-binding activity and LLPS induction, may be essential for CyHV-3 replication.

We addressed this hypothesis by using rational protein engineering based on two complementary observations. First, it has been reported previously that the expression of Zα domains as tandem repeats increases their binding affinity to Z-nucleic acid, as well as their B-to-Z conversion rate (58,59). Second, in proteins containing two Zα domains, these tandem domains are joined by a linker containing charged amino acid residues and an IDR. Based on these observations, we hypothesized that the expression of Zα domains that were unable to rescue the ORF112 deletion when expressed alone may be able to rescue it when expressed as an artificial homotypic or natural heterotypic pair separated by a charged natural linker (Figure 9D, third and fourth columns). Linker sequences were derived from the sequence of the protein from which the first Zα domain was derived. Remarkably, all the good-converter Zα domains tested (HsZα_ADAR1_, HsZα-2_ZBP1_, DrZα-1_PKZ_ and CaZα-1_PKZ_) were able to rescue the ORF112 deletion when expressed as homotypic tandem repeats joined by a linker. In contrast, neither of the two poor-converter Zα domains tested (HsZα-1_ZBP1_ and CaZα-2_PKZ_) were able to rescue the ORF112 deletion when expressed as a homotypic pair. The conversion ability of DrZα-2_PKZ_ has not been reported yet. The testing of natural heterotypic tandem repeats of Zα domains also led to interesting results (Figure 9D, right column). Although none of the Zα domains of Hs ZBP1 and Dr PKZ were capable of rescuing the ORF112 deletion when expressed alone, the heterotypic pairs were capable of doing so. These results suggested that these natural combinations of Zα domains expressed the three functional properties of the ORF112 Zα domain, namely Z-binding activity, LLPS, and B-to-Z conversion. To control the ability of Zα domain tandem repeats able to rescue ORF112 deletion to perform LLPS, three tandem constructs (2 able to rescue the ORF112 deletion and 1 unable to rescue the deletion, see Figure 9D, black circles) were selected for the testing. These Zα domain tandem constructs were expressed as mCherry N-terminal tagged proteins, purified, and tested for their ability to express LLPS (Figure 9E). The results obtained further supported the hypothesis that only Zα domains or combination of Zα domains able to induce LLPS can rescue CyHV-3 ORF112 deletion. First, the two Zα domain constructs able to rescue the ORF112 deletion induced LLPS. Second, the homotypic HsZα-1_ZBP1_ tandem repeat that failed to rescue ORF112 deletion induced only limited level of LLPS.

To further investigate the importance of A-to-Z(RNA)/B-to-Z(DNA) conversion in the CyHV-3 replication cycle, we took advantage of a recent publication reporting the transformation of the non-converter E3 Zα domain of Vv (VvZα_E3_) into a good-converter chimeric molecule by replacing the α3 helix of the former by the α3 helix of HsZα_ADAR1_ (shown to be a good-converter) (18). The resulting chimeric VvZα_E3_α3_HsADAR1_ domain expressed 78% of the B-to-Z transition activity observed for the donor HsZα_ADAR1_ molecule (18). The ability of the VvZα_E3_ and VvZα_E3_α3_HsADAR1_ domains to rescue CyHV-3 ORF112 deletion was then tested by expressing these proteins as isolated Zα domains (Figure 9F, first column), Zα domains fused to the C-terminal end of the CyHV-3 ORF112 N-terminal domain containing an IDR (Figure 9F, second column), and homotypic tandem Zα domains joined by the linker that connects the two Zα domains of Hs ZBP1. Only the homotypic tandem construct of the chimeric VvZα_E3_α3_HsADAR1_ succeeded in rescuing the ORF112 deletion. Remarkably, the control molecule based on VvZα_E3_ did not have this effect. These results confirm *in cellulo* the earlier observation that the structural flexibility conferred by the charge to the α3 helix of HsZα_ADAR1_determines its conversation ability (the linear net charge per residue profile of the two homotypic tandem molecules are presented in Figure 9G). These results suggested that CyHV-3 replication requires a conversion level above a certain minimum.

The CyHV-3 model described in this study provides a unique platform to investigate *in cellulo* the impact of structural changes on the B-to-Z conversion property of a molecule. We aimed to investigate this view by addressing a key step of the B-to-Z conversion process, namely the initial binding of the Zα domain to non-Z-conformed nucleic acids. This interaction has been shown to rely on the structural flexibility of certain Zα domains, such as HsZα_ADAR1_, or on positive charges in the β-wing that mediate interactions with other nucleic acid conformations, such as B-DNA (19,60). However, alignment of all the Zα domains tested in this study did not lead to the identification of conserved positively charged amino acids strictly correlating with conversion ability (Supplementary Figure S5). Using *in silico* rational design, we hypothesized that replacing the last six residues of VvZα_E3_ by arginine (R) residues (hereafter called VvZα_E3_6R) might confer the conversion property and thus also the ability to rescue the ORF112 deletion. The ability of the VvZα_E3_6R domain to rescue the ORF112 deletion was tested when expressed as isolated Zα domain (Figure 9F, first column), as Zα domain fused to the C-terminal end of the ORF112 N-terminal domain containing an IDR (Figure 9F, second column), and as homotypic tandem Zα domain repeats joined by the linker connecting the two Zα domains of Hs ZBP1 (Figure 9F, third column, Figure 9G, lower panel). Interestingly, the last of these (VvZα_E3_6R-L-VvZα_E3_6R) rescued the ORF112 deletion, whereas the WT molecule (VvZα_E3_-L-VvZα_E3_) did not.

Together, these results demonstrated that the ORF112 Zα domain is essential for viral replication and that it forms dynamic RNPs in infected cells. Rescuing of the ORF112 deletion required the expression of a Zα domain-containing amino acid sequence that combined three properties: Z-binding activity, LLPS induction, and A-to-Z conversion. Each of these properties was essential but not sufficient for viral growth. Only certain Zα domains were able to rescue the ORF112 deletion when expressed in isolation (Figure 9A). However, the natural combination of heterotypic tandem Zα domains joined by a charged linker was able to rescue the ORF112 deletion (Figure 9D). In the final section of this study, we selected one prominent example of such natural combinations (Hs ZBP1) to study the involvement of the protein in the formation of dynamic RNP condensates in CyHV-3 infected cells.

### The heterotypic Zα domain tandem repeat of Hs ZBP1 accumulates in dynamic RNP condensates in CyHV-3 infected cells

**Figure 10.**
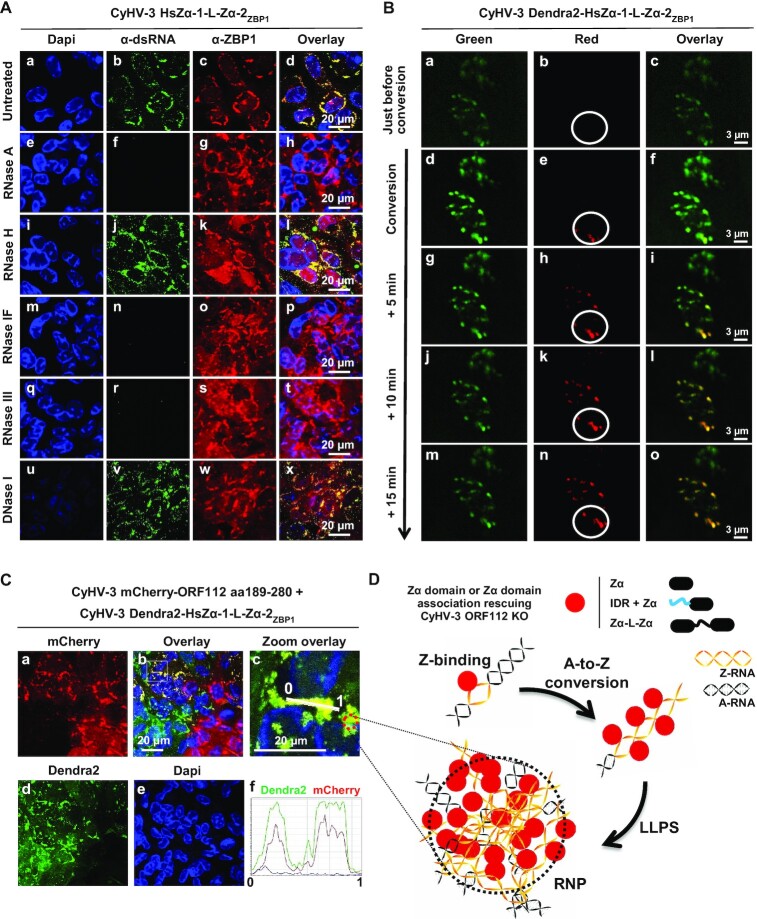
The heterotypic Zα domain tandem repeat of Hs ZBP1 accumulates in dynamic RNP condensates in CyHV-3 infected cells. (**A**) Cells infected with the CyHV-3 HsZα1-L-Zα2_ZBP1_ recombinant expressing the heterotypic Zα domain tandem repeat of Hs ZBP1 instead of ORF112 were fixed, permeabilized, and treated with the indicated enzymes before immunostaining of dsRNA (α-dsRNA, green signal) and ZBP1 (α-ZBP1, red signal). DNA was stained with Dapi (blue signal). (**B**) Living cells infected with the CyHV-3 Dendra2-HsZα1-L-Zα2_ZBP1_ recombinant expressing the fluorescence Dendra2 protein fused at its C-terminal end to the heterotypic Zα domain tandem repeat of Hs ZBP1 were submitted to green-to-red photoconversion on the indicated ROI (FLAP experiment). Representative pictures collected according to time are presented. (**C**) CCB cells were coinfected with the indicated CyHV-3 recombinant strains (MOI of 1 PFU/cell for each recombinant). Analysis of coinfected cells revealed that mCherry-ORF112 aa189–280 and Dendra2-HsZα1-L-Zα2_ZBP1_ colocalize in RNP condensates. (**D**) Schematic model linking biochemical properties of Zα domains and the formation of RNP observed in CyHV-3 infected cells.

The ability of the natural heterotypic Zα domain tandem repeat of Hs ZBP1 to rescue the ORF112 deletion suggested that this protein would exhibit a subcellular distribution and a mobility in CyHV-3 infected cells that are comparable to those observed for the CyHV-3 ORF112 Zα domain (Figures 4 and 6C). This hypothesis was investigated using the approaches described above. Immunostaining of Hs ZBP1 in CCB cells infected by the CyHV-3 HsZα1-L-Zα2_ZBP1_ recombinant virus revealed a subcellular localization comparable to that of ORF112 Zα domain and a colocalization with dsRNA (Figure 10A). We then produced a recombinant strain of CyHV-3 expressing HsZα1-L-Zα2_ZBP1_ as a Dendra2 fusion protein, in order to study the mobility of this protein using photoconversion and time-lapse video confocal microscopy in living infected cells (Figure 10B). These analyses showed that the natural heterotypic Zα domain tandem repeat of Hs ZBP1, like the CyHV-3 ORF112 Zα domain, moved rapidly between RNP condensates. Finally, to compare further the subcellular localization of HsZα1-L-Zα2_ZBP1_ and the ORF112 Zα domain expressed during viral infection, CCB cells were coinfected with a mixture of CyHV-3 recombinants expressing either Dendra2-HsZα1-L-Zα2_ZBP1_ or the mCherry-ORF112 Zα domain (Figure 10C). Confocal analysis revealed cells infected by only one of the two recombinants and cells coinfected by both (Figure 10C, panels a, b, and d). Interestingly, analysis of Dendra2 and mCherry fluorescence distribution in the later cells revealed a perfect colocalization of the two signals (Figure 10C, panels c and f).

## DISCUSSION

The Zα domain protein family consists of multidomain cellular and viral proteins that are involved in regulating innate immunity. The few studies that have investigated the exchangeability of Zα domains between Zα domain-containing proteins have suggested that Zα domains are functional homologs. This impression has been strengthened by NMR and crystallographic studies (11,60) demonstrating that different Zα domains bind similarly to Z-DNA. However, biochemical experiments performed with dsDNA (B-DNA and Z-DNA) have highlighted the functional diversity of Zα domains in terms of dsDNA recognition (B *versus* Z) and B-to-Z conversion (17–19). Because this functional diversity has been studied only in cell-free assays and never in intact cells, the biological relevance of A-to-Z (Z-RNA)/B-to-Z (Z-DNA) conversion (9,10,23,24) still need to be addressed using biological models. Here, we studied the Zα domain-containing protein ORF112 of CyHV-3. Genome editing of CyHV-3 demonstrated that expression of only the Zα domain was sufficient for normal virus replication in cell culture and virulence in carp. In contrast, deletion of the Zα domain was lethal for the virus. These observations revealed the potential of the CyHV-3 model as a unique platform for comparing the exchangeability of Zα domains expressed alone in living cells. Rescue of the CyHV-3 ORF112 deletion required the expression of a Zα domain-containing sequence that combined three properties: Z-binding activity, LLPS induction, and A-to-Z conversion. Each of these properties was essential but not sufficient for viral growth. The ORF112 Zα domain and certain Zα domains able to rescue the ORF112 deletion were found to accumulate in dynamic RNPs in CyHV-3 infected cells.

Our work is the first to report the ability of certain Zα domains to induce LLPS. This fascinating biological process has never been reported for Zα domains. Moreover, the finding that all Zα domains do not express this property unravelled a new level of functional diversity among Zα domains. Thus, LLPS induction was intrinsic of a few single Zα domains with a high predicted charge (Figure 9B). This observation further supports the recognized importance of positive charge as a feature of LLPS inducer proteins (53,61). We also found that other Zα domains that were unable to rescue the ORF112 deletion when expressed alone acquired this property when linked to LLPS inducer polypeptides, such as the N-terminal domain of ORF112 (Figure 9D, HsZα_ADAR1_ and HsZα-2_ZBP1_) or natural intrinsically disordered linkers present between Zα domains in cellular proteins (Figure 9D, artificial homotypic Zα domain repeats made of HsZα_ADAR1_, HsZα-2_ZBP1_, DrZα-1_PKZ_ and CaZα-1_PKZ_, and natural heterotypic Zα domain repeats of Hs ZBP1 and Dr PKZ). It is notable that all cellular Zα domain-containing proteins possess at their N-terminus two tandem Zα domains (except for Hs ADAR1, for which the Zα domain is associated with a Zβ domain, in contrast to teleost ADAR1 orthologues, which contain two Zα domains). The two Zα domains are separated systematically by an intrinsically disordered linker of conserved size but divergent sequence (62). The only known exception is ADAR1, where the linker is twice as long and consists of two nearly identical copies of a peptide (63,64). The intrinsic ability of some Zα domains to induce LLPS and the association of Zα domains (independently of their inherent ability to induce LLPS) with LLPS inducer polypeptides (IDR encoded by the N-terminus of CyHV-3 ORF112 and the inter-Zα domain linker of cellular proteins) suggest that LLPS induction associated with Zα domains has been subject to convergent evolution. The discovery of this new property associated with Zα domains will stimulate new hypotheses about the mechanisms of action and function of proteins containing Zα domains. It supports interesting hypotheses described in a recent review (65) on the mechanism of action of Zα domain containing proteins. The induction of LLPS could induce interactions with other proteins or could contribute to A-to-Z (Z-RNA)/ B-to-Z (Z-DNA) conversion by Zα domains (see below).

The CyHV-3 model highlights in living cells the biological relevance of A-to-Z (Z-RNA)/ B-to-Z (Z-DNA) conversion mediated by Zα domains. All Zα domains exhibit the property of binding with high affinity to Z-DNA and Z-RNA, leading to stabilization of the Z conformation. In addition, some Zα domains can bind to Z-prone B-DNA/A-RNA sequences and induce B-to-Z (Z-DNA)/A-to-Z (Z-RNA) conversion. This functional diversification among Zα domains has depended on cell-free biochemical experiments that led to the classification of Zα domains as non-converters, poor-converters, and good-converters. Conversion mediated by Zα domains is described as a multi-step process. The first critical step represents the binding of a Zα domain to one side of the Z-prone B-DNA/A-RNA sequence (18,66,67). This binding initiates the conversion of the nucleic acids to the Z-form and promotes the binding of a second Zα molecule, stabilizing the Z-conformation (66–68).

Our study indicates *in cellulo* the biological relevance of A-to-Z conversion and supports several *in vitro* observations related to conversion mediated by the Zα domain. First, we observed that all Zα domains (whether expressed alone or in association with other domains) containing sequences able to rescue the ORF112 deletion were good-converters (Figure 9). Second, a recent study based on rational protein engineering demonstrated *in vitro* the possibility of transforming the non-converter E3 Zα domain into a good-converter by replacing the α3 helix of Vv E3 by the α3 helix of HsZαADAR1 (18). In contrast to the parental E3 Zα domain, the resulting chimeric Zα domain could rescue the ORF112 deletion when expressed as a homotypic Zα domain tandem repeat (Figure 9F). Third, the positive charge in the β-wing of certain Zα domains has been shown to affect positively their ability to interact with other nucleic acid conformations than Z and promote subsequent conversion (19,60). Thus, the non-converter Zα domain of Vv E3 acquired the ability to convert B-DNA to Z-DNA when the amino acid residues in its β-wing region were substituted by polar or positively charged residues (19). Supporting this *in vitro* observation, we showed that substituting the last six residues of Vv E3 Zα by six arginine residues allowed this mutated Zα domain to rescue the ORF112 deletion when expressed as a homotypic tandem repeat (Figure 9F). Polar and positively charged residues such as arginine and lysine typify intrinsically disordered proteins. Consequently, these residues might also promote LLPS through charge-charge interactions (69,70). Future investigations could usefully include determining whether induction of LLPS by Zα domains or associated domains positively affects the conversion mediated by Zα domains. Fourth, it has been proven that a converter Zα domain exhibited the greatest propensity to induce conversion when tethered to another Zα domain (58). It has been proposed that dimerization increases conversion efficiency due to the stabilization induced by fixation of the second Zα domain in the newly formed Z-nucleic acid. Further supporting the biological relevance of Z-conversion mediated by Zα domains, we observed that some good-converter Zα domains that were unable to rescue the ORF112 deletion when expressed alone or fused to the ORF112 N-terminal domain were able to do so when expressed as homotypic or heterotypic Zα domain tandem repeats (Figure 9D). Matteo de Rosa *et al.* proposed a model for the interaction of multiple Zα domains with dsDNA/dsRNA (71). This model suggests successive steps of conversion and stabilization of the Z-DNA conformation and predicts the left-handed conformation propagation from a starting nucleation point. A long and perfectly alternating purine/pyrimidine sequence with a propensity for Z-conformation prone is expected to accumulate in a proteinaceous aggregate with Z-DNA similar to the RNPs we observed in CyHV-3 infected cells. An identical oligomerization process on a substrate has been observed previously for the RNA sensor MDA5 (72). Future investigations may include determining whether induction of LLPS by Zα domains or associated IDRs can contribute to nucleation along a Z-prone nucleic acid.

Z-binding, A-to-Z RNA conversion, and LLPS induction may form part of an immune evasion mechanism developed by CyHV-3. The initial description of the Z-binding activity of the CyHV-3 ORF112 Zα domain, together with the demonstration using gel mobility assay that this domain was able to outcompete the binding of cellular PKZ to Z-DNA, led to the hypothesis that ORF112 may be an immune evasion protein. According to this hypothesis, ORF112 binding to Z-DNA/Z-RNA would prevent their detection by Zα domains of the innate immune system (ADAR1 and PKZ in cypriniforms; teleosts do not encode a ZBP1 orthologue). Supporting this hypothesis, a recent study has unravelled a mechanism by which the Zα domain of Vv E3 inhibits ZBP1-dependent necroptosis in IFN-stimulated cells (73). In the context of such a competition mechanism, it would be expected that the ORF112 Zα domain could be replaced by any Zα domain expressing Z-binding activity. Our data indicated that only amino acid sequences encompassing Z-binding activity, LLPS induction, and A-to-Z conversion could rescue ORF112 deletion, thus suggesting a more complex mechanism. Despite the fact that herpesviruses have dsDNA genomes, their replication cycle has been associated with the expression of cytoplasmic dsRNA of unknown sequence (45,74). Our work is the first to confirm this phenomenon for a member of the family *Alloherpesviridae* (Figures 4A and 10A). The expression of dsRNA in herpesvirus infected cells exposes the virus to antiviral cellular proteins expressing dsRNA-binding domains, such as PKR, RIG-I, and MDA5. In the context of previous findings, our work prompts an original model of immune evasion mediated by CyHV-3 Zα domain (Figure 10D). According to this model, the binding of a good-converter Zα domain to Z-RNA and to Z-prone A-RNA sequences leads to the stabilization of Z-RNA and A-to-Z conversion, respectively. A-to-Z conversion then progresses from these initial Z-binding sites through oligomerization on the substrate. Together with the induction of LLPS, this phenomenon contributes to the formation of RNP condensates, thereby preventing the detection of Z-RNA and dsRNA by their respective cellular sensors. Future work will focus on identifying the sequences of RNA molecules and proteins involved in the observed RNP condensates. Proximity labelling experiments using Apex2-tagged ORF112 recombinant proteins are in progress to reach this goal.

Both Zα domain containing proteins Vv E3 (6) and CyHV-3 ORF112 (Figure 4A) are involved in the formation of RNP condensates in infected cells. The functional diversity observed between the Zα domains of Vv E3 and CyHV-3 ORF112 suggests that the formation of condensates by these viral proteins relies on different mechanisms acquired through convergent evolution. On one hand, the Zα domain of CyHV-3 could induce RNP condensates through charge based-LLPS formation; on the other hand, the Zα domain of Vv E3 (unable to induce LLPS) could induce RNP condensates through its association with the dsRNA binding domain contained in the protein.

Various observations suggest that the conversion of nucleic acid conformation may be a general mechanism for regulating the interactions of dsDNA and dsRNA with cellular sensors. Our observation that the ORF112 deletion was rescued by cellular Zα domains (expressed alone or in tandem repeat) suggests that the property expressed by CyHV-3 as a potential immune evasion mechanism is also exploited in cell biology. Thus, we showed that the natural heterotypic Zα domain tandem repeat of Hs ZBP1 induced RNP in CyHV-3 infected cells in a manner identical to that of the CyHV-3 ORF112 Zα domain (Figure 10). Our study also opens new perspectives for investigation of Zα domain-dependent suppression of dsRNA detection. For example, future investigations could involve determining whether the good-converter Zα domain of ADAR1p150 (11) is able to antagonize PKR (75,76), RIG-I (77) and MDA5 (78) by converting A-RNA to Z-RNA, thereby making these RIG-I-like receptors blind to sensing dsRNA. Future investigations could also involve determining whether some of the mutations inducing Aicardi-Goutières syndrome (79) are associated with a defect of conversion rather than a defect of Z-binding activity. Further supporting the importance of the conversion of nucleic acid conformation as a general mechanism to regulate the interactions of dsDNA and dsRNA with cellular proteins, a recent study reported the ability of the bacterial DNA-binding protein DNABII to reduce NET-mediated bacteria killing through the conversion of polymorphonuclear neutrophile-released B-DNA to Z-DNA (80).

In conclusion, our study presents a biochemical and biological characterization of a broad spectrum of Zα domains using a unique model. For the first time, we report the ability of Zα domains to induce LLPS and demonstrate the biological relevance of A-to-Z conversion. Our study also confirms and expands the functional diversity of Zα domains described previously *in vitro*. Importantly, it opens new perspectives on the roles of Zα domains as negative regulators of dsRNA detection by cellular sensors.

## DATA AVAILABILITY

The software and algorithms used in this study are listed in Supplementary Table S3 provided as supplementary materials.

## Supplementary Material

gkac761_Supplemental_Files
