# Supplementary material for: A fish herpesvirus highlights functional diversities among Zα domains related to phase separation induction and A-to-Z conversion
Source: Nucleic Acids Res. 2022 Sep 22;51(2):806–30. doi: 10.1093/nar/gkac761 (PMC9881149; doi:10.1093/nar/gkac761)
Supplement: gkac761_Supplemental_Files [file gkac761_supplemental_files.zip › 07182022 Diallo et al. Supplementary material legends.docx]

**SUPPLEMENTARY MATERIAL LEGENDS**

**Table S1. Synthetic nucleic acid oligonucleotides used in this study.**

**Table S2. Amino acid sequence of the proteins tested in this study.**

**Table S3. Software and algorithms used in this study.**

**Figure S1. Testing of ORF112 essentiality for viral growth in cell culture using a coinfection‑based approach with a helper virus.** (A) Flowchart of the experiment. CCB cells were cotransfected with plasmids CyHV-3 BAC ORF149 KO (deleted for non-essential ORF149 but encoding WT ORF112) and CyHV‑3 BAC ORF112 KO (deleted for essential ORF112 but encoding WT ORF149). Immunophenotyping of the transfected cells was performed 48 h posttransfection using α‑ORF112 (red signal) and α‑ORF149 (pink signal) antibodies. Cell supernatant was collected 72 h posttransfection and used to infect fresh CCB cell monolayers, which were immunostained with α‑ORF112 and α‑ORF149 antibodies at 24 and 48 h postinfection. (B) Illustration of the phenotypes observed, based on ORF112 and ORF149 expression for transfected (left column) and infected (middle and right columns) cells. Each cluster of four photographs represents the analysis of the same specimen for EGFP (green signal, lower left; the BAC cassette encodes EGFP), ORF112 (red signal, upper left), and ORF149 (pink signal, upper right). The lower right photographs represent the overlay of these three signals and DAPI. The percentage indicated in the overlay panel represents the relative abundance of the phenotype among all phenotypes observed, based on observation of 300 randomly selected EGFP cells or cell clusters. The efficiency of cotransfection was controlled by immunostaining with α‑ORF112 and α‑ORF149 antibodies, which revealed doubly positive cells (panels a-d). Virions resulting from cotransfection were collected from cell supernatants, diluted, and inoculated onto CCB monolayers. Double immunofluorescent staining of the monolayers 24 h postinfection revealed three phenotypes. The major phenotype (75%) consisted of ORF112/ORF149 doubly positive cells representing the emergence of WT recombinant. The second most frequent phenotype (24%) consisted of singly deleted ORF149. Finally, 1% of EGFP-fluorescent cells expressed ORF149 but were negative for ORF112. These cells represented infection by virions carrying the ORF112-deleted genotype. Cells expressing EGFP but negative for ORF112 and ORF149 were not detected. Immunofluorescent staining of cell monolayers 2 days postinfection revealed plaques that were doubly positive and singly positive for ORF112. Neither isolated cells nor plaques negative for ORF112 were observed, suggesting that infection by ORF112-negative virions detected 24 h postinfection led to abortive infection or early death of infected cells. These data confirmed that ORF112 is essential for CyHV-3 growth in cell culture.

**Figure S2. Controls of LLPS experiments.** This figure is related to Figure 8 (panels B and C). LLPS was induced by incubation of the indicated purified recombinant proteins (10 µM) with PEG (5%, v/v) (panels A and B) or 10 µM of Z-RNA (panel A) or 10 µM of FAM-Z-RNA (panel B).

**Figure S3. LLPS of mCherry N-terminal tagged ORF112 recombinant proteins induced by PEG.** This figure is related to Figure 8B. LLPS was induced by incubation of the indicated purified recombinant forms of ORF112 (10 µM) and PEG (5%, v/v). (A) Quantitative comparison of several characteristics of PEG‑induced LLPS by the indicated recombinant forms of ORF112. Each point represents the means ± the SEM from independent experiments. (B) Bleaching was performed at the indicated time points after LLPS induction. The recovery of fluorescence was followed over time.

**Figure S4. Representation of global predicted charge *versus* the number of positively charged amino acids of Zα domains.** This figure is an update version of panel B of Figure 9. Three Zα domains encoded by mouse proteins were added to the graph (presented in grey): the two Zα domains of mouse ZBP1 (MmZα-1_ZBP1_ and MmZα-2_ZBP1_) and the unique Zα domain of mouse ADAR1 (MmZα‑1_ADAR1_). In contrast to the other Zα domains presented in this figure, the three mouse Zα domains were not tested in this study for their ability to rescue CyHV-3 ORF112 deletion. Zα domains able or not able to rescue virus replication are presented in blue or black, respectively.

**Figure S5. Charge distribution on the Zα wings of Zα domains.** The structure of CyHV-3 ORF112 Zα domain is shown at the top (12). Zα domains listed in Figure S4 were aligned. The vertical bars above the sequences illustrate the degree of conservation. In contrast to the other Zα domains presented in this figure, the three mouse Zα domains (presented in grey) were not tested in this study for their ability to rescue CyHV-3 ORF112 deletion. Zα domains able or not able to rescue virus replication are presented in blue or black, respectively. Positive residues in the β2- β-wing- β3 region are highlighted in red.

**Movie S1.** Time-lapse of LLPS induced by incubation of mCherry N-terminal tagged ORF112 protein. This movie is related to Figure 8B. LLPS was induced by incubation of the purified protein (10 µM) with PEG‑6000 (5%, v/v). Droplet formation was observed by confocal microscopy. For evaluation of morphological changes, the volume and surface softness were processed by Imaris software using surface building function. The total length of the movie represents 50 minutes of real‑time starting 1 minute after LLPS induction.

**Movie S2.** Coalescence of phase-separated droplets formed by mCherry N-terminal tagged ORF112. This movie is related to Figure 8B. The movie is made by converting multiple z-tack of an on-going LLPS using Leica Application Suite Advanced Fluorescence (LAS-AF) software. The total length of the movie represents 3.5 minutes of real‑time starting 30 minutes after LLPS induction.
