# Supplementary material for: A fish herpesvirus highlights functional diversities among Zα domains related to phase separation induction and A-to-Z conversion
Source: Nucleic Acids Res. 2022 Sep 22;51(2):806–30. doi: 10.1093/nar/gkac761 (PMC9881149; doi:10.1093/nar/gkac761)
Supplement: gkac761_Supplemental_Files [file gkac761_supplemental_files.zip › Figure S1 revised version 07142022.pptx]

## Slide 1
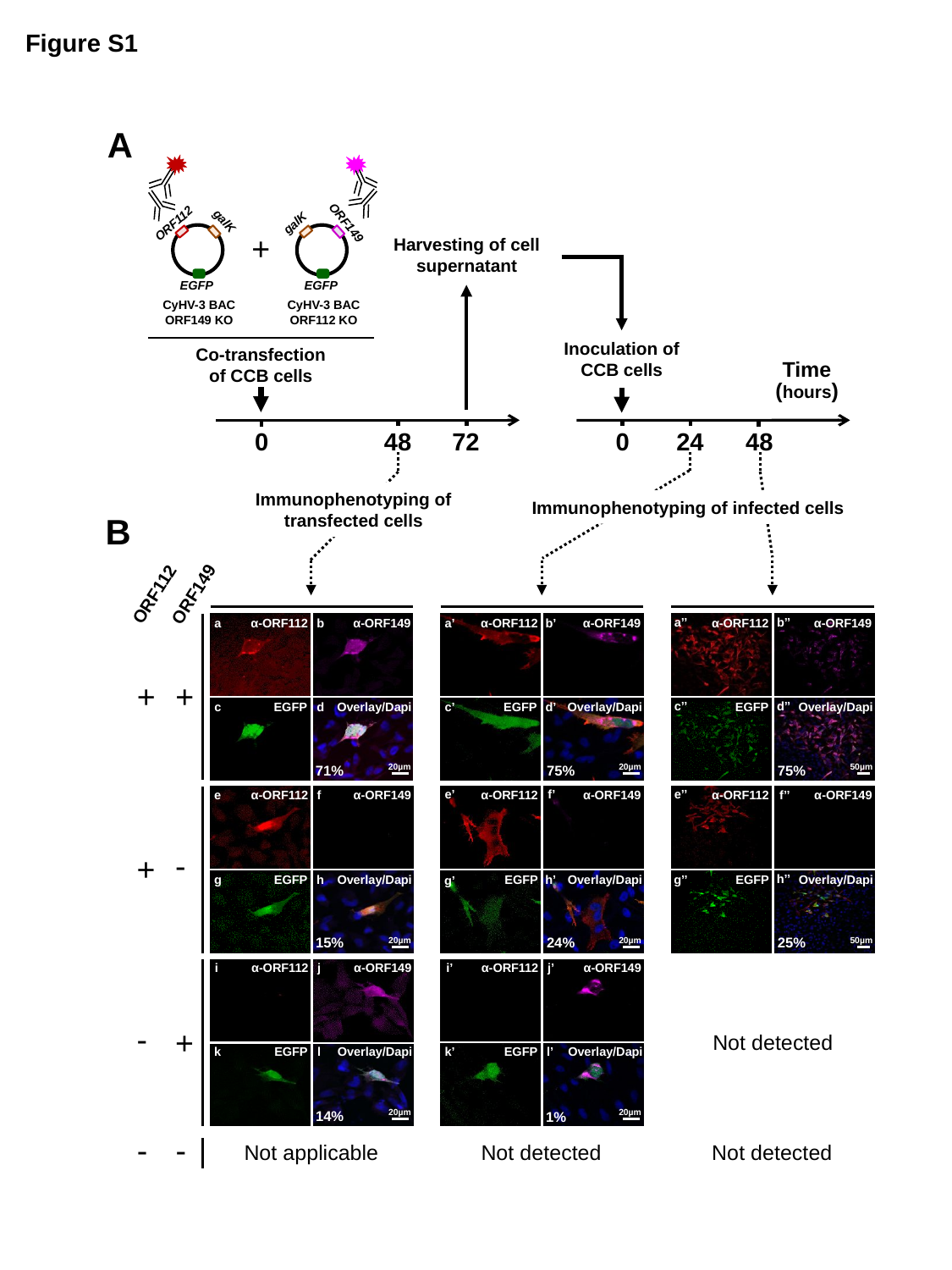

Figure S1
A
galK
ORF149
galK
ORF112
EGFP
EGFP
+
Harvesting of cell supernatant
CyHV-3 BAC ORF149 KO
CyHV-3 BAC ORF112 KO
Inoculation of CCB cells
Co-transfection of CCB cells
Time (hours)
0
24
48
0
48
72
Immunophenotyping of transfected cells
Immunophenotyping of infected cells
B
ORF112
ORF149
a
b
α-ORF149
c
d
Overlay/Dapi
α-ORF112
α-ORF112
α-ORF149
Overlay/Dapi
α-ORF112
α-ORF149
Overlay/Dapi
+
+
EGFP
EGFP
EGFP
 50µm
 20µm
 20µm
e
f
α-ORF149
g
h
Overlay/Dapi
α-ORF112
α-ORF112
α-ORF149
Overlay/Dapi
α-ORF112
α-ORF149
Overlay/Dapi
-
+
EGFP
EGFP
EGFP
 50µm
 20µm
 20µm
i
α-ORF112
j
α-ORF149
α-ORF112
α-ORF149
-
+
Not detected
k
EGFP
l
Overlay/Dapi
EGFP
Overlay/Dapi
 20µm
 20µm
-
-
Not applicable
Not detected
Not detected
b’’
a’’
b’
a’
d’’
c’’
d’
c’
 75%
 75%
 71%
f’
e’
e’’
f’’
h’’
h’
g’’
g’
 25%
 15%
 24%
j’
i’
k’
l’
 14%
 1%
