# Supplementary material for: A fish herpesvirus highlights functional diversities among Zα domains related to phase separation induction and A-to-Z conversion
Source: Nucleic Acids Res. 2022 Sep 22;51(2):806–30. doi: 10.1093/nar/gkac761 (PMC9881149; doi:10.1093/nar/gkac761)
Supplement: gkac761_Supplemental_Files [file gkac761_supplemental_files.zip › Figure S2 revised version 07142022.pptx]

## Slide 1
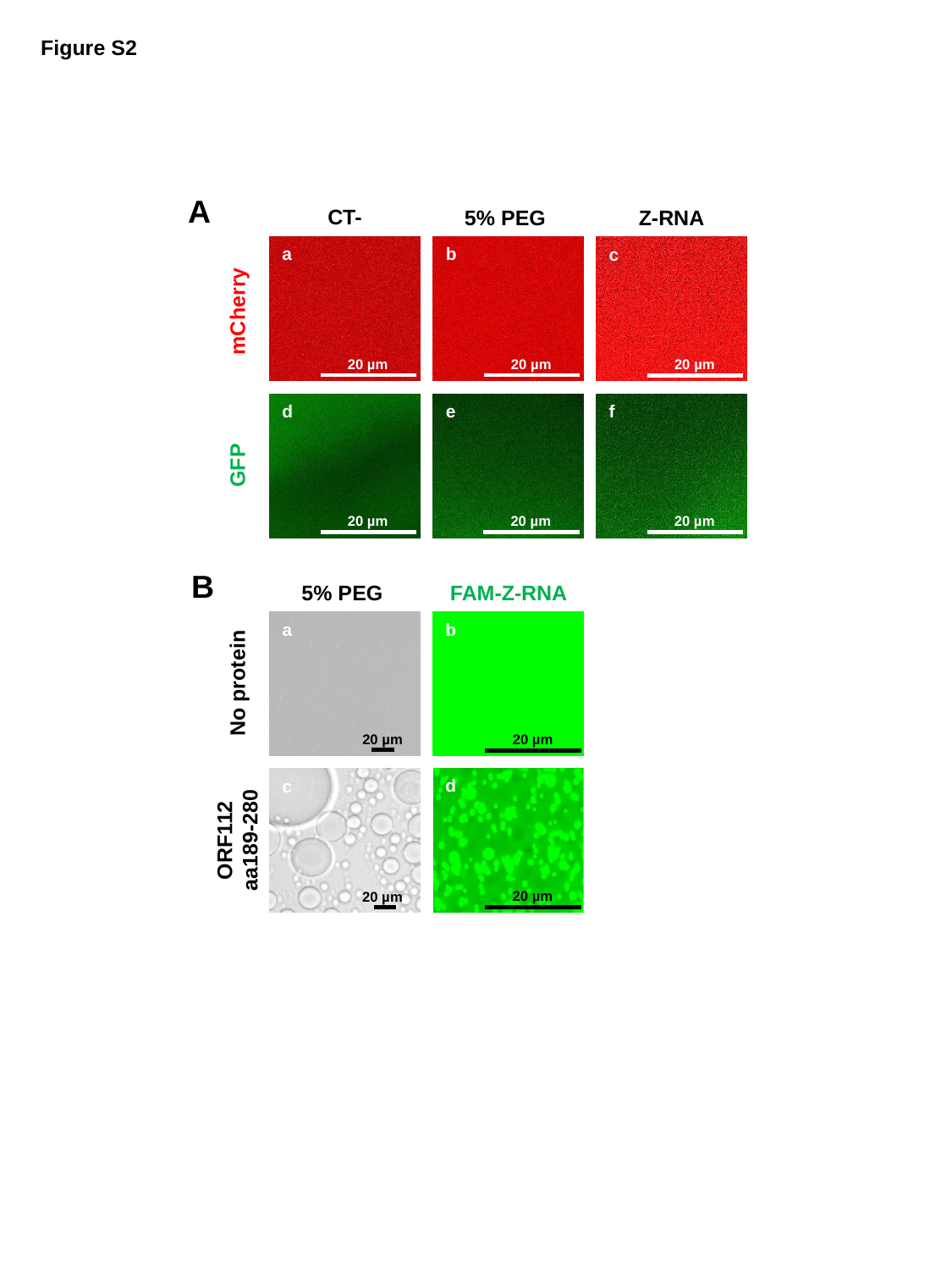

Figure S2
A
CT-
5% PEG
Z-RNA
a
b
c
mCherry
20 µm
20 µm
20 µm
d
e
f
GFP
20 µm
20 µm
20 µm
B
FAM-Z-RNA
b
20 µm
d
20 µm
5% PEG
a
No protein
20 µm
c
 ORF112
aa189-280
20 µm
20 µm
