# Supplementary material for: A fish herpesvirus highlights functional diversities among Zα domains related to phase separation induction and A-to-Z conversion
Source: Nucleic Acids Res. 2022 Sep 22;51(2):806–30. doi: 10.1093/nar/gkac761 (PMC9881149; doi:10.1093/nar/gkac761)
Supplement: gkac761_Supplemental_Files [file gkac761_supplemental_files.zip › Figure S3 revised version 07142022.pptx]

## Slide 1
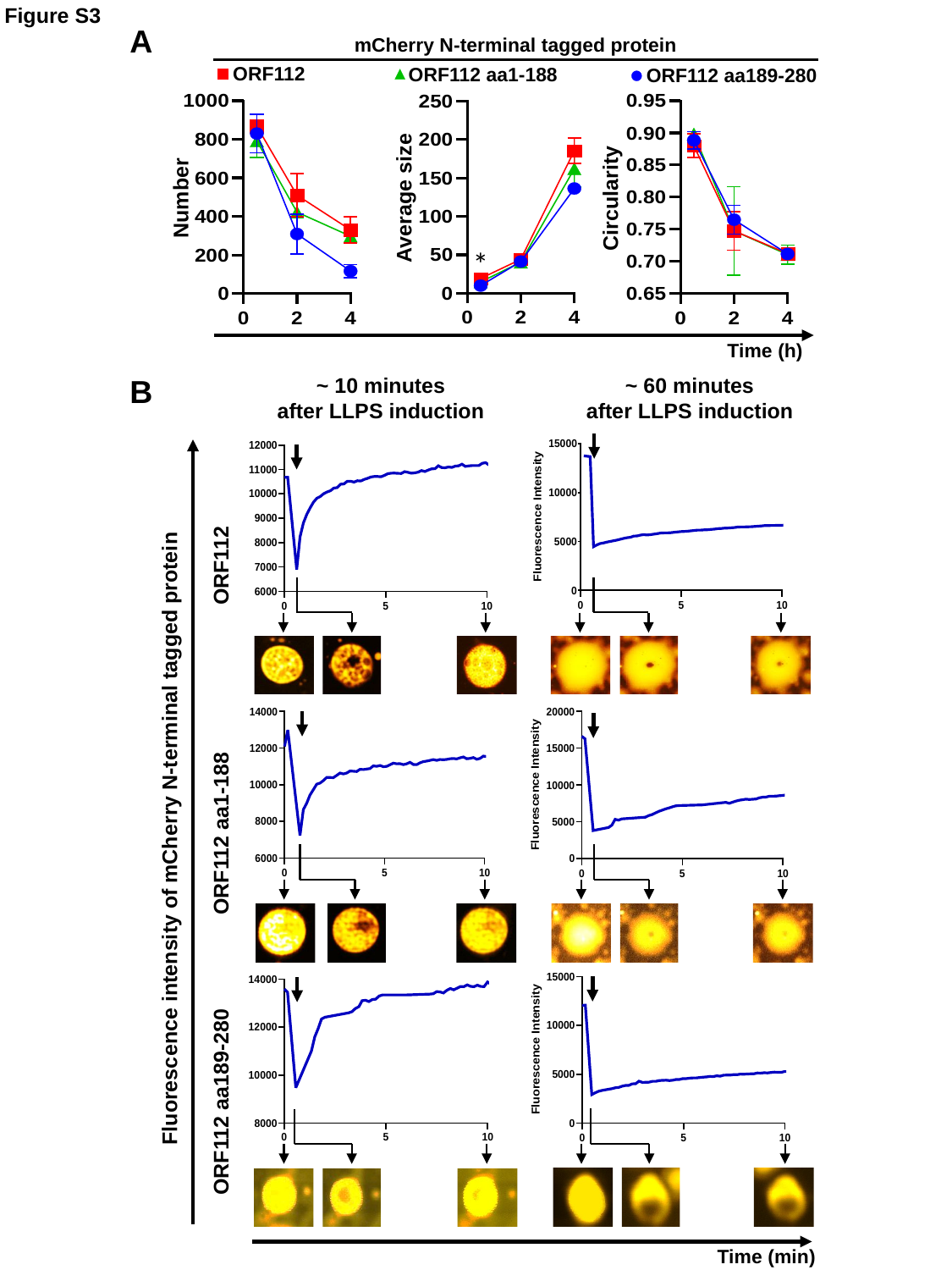

Figure S3
A
mCherry N-terminal tagged protein
ORF112 aa189-280
ORF112
ORF112 aa1-188
Number
Average size
Circularity
*
Time (h)
~ 10 minutes
after LLPS induction
~ 60 minutes
after LLPS induction
ORF112
B
ORF112 aa1-188
Fluorescence intensity of mCherry N-terminal tagged protein
ORF112 aa189-280
Time (min)
