# Supplementary material for: A fish herpesvirus highlights functional diversities among Zα domains related to phase separation induction and A-to-Z conversion
Source: Nucleic Acids Res. 2022 Sep 22;51(2):806–30. doi: 10.1093/nar/gkac761 (PMC9881149; doi:10.1093/nar/gkac761)
Supplement: gkac761_Supplemental_Files [file gkac761_supplemental_files.zip › Figure S4 revised version 07142022.pptx]

## Slide 1
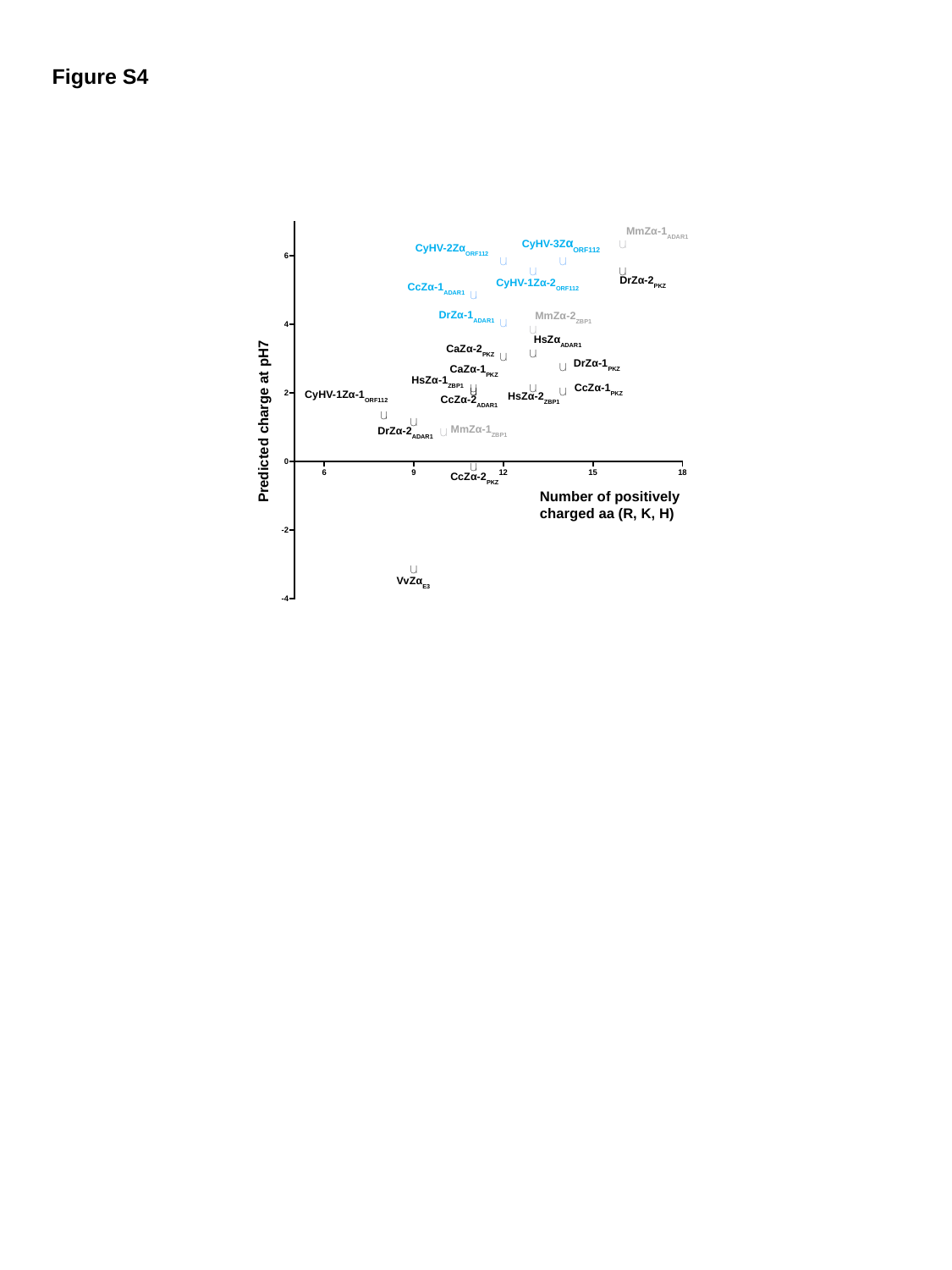

Figure S4
MmZα-1ADAR1
CyHV-3ZαORF112
CyHV-2ZαORF112
DrZα-2PKZ
CyHV-1Zα-2ORF112
CcZα-1ADAR1
DrZα-1ADAR1
HsZαADAR1
CaZα-2PKZ
DrZα-1PKZ
CaZα-1PKZ
HsZα-1ZBP1
CcZα-1PKZ
CyHV-1Zα-1ORF112
HsZα-2ZBP1
CcZα-2ADAR1
DrZα-2ADAR1
CcZα-2PKZ
VvZαE3
MmZα-2ZBP1
Predicted charge at pH7
MmZα-1ZBP1
Number of positively charged aa (R, K, H)
